# Social inclusion policy effects on democratic satisfaction in Europe: a catalyst of polarization threating the identities of privileged social groups

**DOI:** 10.3389/fsoc.2025.1567394

**Published:** 2025-06-04

**Authors:** Ibrahim Olayinka Akinyemi, Martin Groß, Volker Lang

**Affiliations:** Institute of Sociology, University of Tübingen, Tübingen, Germany

**Keywords:** democratic satisfaction, inclusive policies, religious freedom, migrant integration, xenophobia, homophobia, gender equality, country comparison

## Abstract

This study analyses the influence of inclusive policies on the democratic satisfaction of different social groups. It draws on social identity theory to explain how inclusive policies can contribute to conflicts and polarization in attitudes between social groups. More specifically, inclusive policies aim to improve the rights and social recognition of disadvantaged groups while they reduce the privileges of groups traditionally recognized as superior. Consequently, we expect that democratic institutions (as the providers of these policies) either get support or disproval from the respective social groups for inclusive policies—causing related increases and decreases in the democratic satisfaction of the respective groups. Using longitudinal data from European Social Survey (rounds 1–10) and additional country level data, we test how socially inclusive policies affect differences in democratic satisfaction between disadvantaged and privileged groups in four policy areas: (1) religious freedom, (2) inclusion of migrants, (3) equal treatment of homosexuals, and (4) gender equality. Except for gender equality policies, our findings support our hypothesis: socially inclusive policies lead to less democratic satisfaction for groups traditionally recognized as superior while the democratic satisfaction of formerly disadvantaged groups increases. These changes in democratic satisfaction indicate that inclusive policies lead to gains in equal social recognition (Isothymia) for some but at the same time are considered a threat to privileged social recognition (Megalothymia) by others. With respect to support for (democratic) political institutions inclusive policies are therefore a “double-edged sword” and need to be implemented with care. However, given comparatively strong inclusive policies regarding religious freedom and migrant integration our analyses also indicate convergence in democratic satisfaction between disadvantaged and privileged social groups.

## 1 Introduction

It is undeniable that people's support for democratic institutions is essential for maintaining social cohesion. However, democracies in Europe, as elsewhere, are facing declining vertical social cohesion, i.e., decreases of trust in institutions and democratic satisfaction (e.g., Anderson and Guillory, 1997; Reichert, 2016). This decline in popular legitimacy of democracy may consequently give rise to authoritarian leadership (Norris and Inglehart, 2019), underpinning the importance to understand what factors affect vertical social cohesion.

Recent research suggests that future studies should take interactions between social groups on the meso-level and macro-level structures into account (Fonseca et al., 2019). This study targets at this meso-macro relationship by investigating how the democratic satisfaction of potentially conflicting social groups on the meso-level changes in response to political macro-level interventions. Do socially inclusive policies lead to an overall increase in democratic satisfaction, or are they rather a catalyst of ongoing polarization by antagonizing some social groups and decreasing their democratic satisfaction?

Some recent studies point to the fact that many group conflicts result from threatened social identities (Babst et al., 2024): groups with traditionally less social recognition like women, migrants or homosexuals struggle for equal recognition (demands for “Isothymia”, Fukuyama, 2018), while so far superiorly recognized groups like men, natives or heterosexuals may feel threatened by these attempts (demands for “Megalothymia”, Fukuyama, 2018). Socially inclusive policies affect these struggles since they fulfill the demands for Isothymia of disadvantaged groups by changing institutional regulations, which might foster the democratic satisfaction of these groups. At the same time, traditionally superior recognized groups may feel threatened by those inclusive policies as they diminish their “special rights” (Norris and Inglehart, 2019). For those groups, these threats to Megalothymia instill the feeling that their wishes and needs are not respected by political leaders anymore, which in turn might decrease their democratic satisfaction.

In summary, while inclusive policies improve the Isothymia of some social groups they also threaten the megalothymic identity of other groups. Thus, integration politics could lead to very different outcomes: because of the former, they could narrow the gap between disadvantaged and privileged groups, because of the latter they could even lead to increasing differences in democratic satisfaction between social groups, and thus, contributing to polarization. This study aims to analyze the impact of inclusive policies on vertical social cohesion. Therefore, we investigate the influence of inclusive policies on the democratic satisfaction of disadvantaged and privileged social groups in four policy areas: (1) religious freedom, (2) inclusion of migrants, (3) equal treatment of homosexuals, and (4) gender equality.

## 2 Theoretical framework

### 2.1 Social identity and group polarization theories

This study borrows its main theoretical argument from the Social Identity Theory of Tajfel and Turner (1986) who posit that individual's belonging to a social group is important for their personal identity. The quest for a positively evaluated identity is a primary need of human beings and forms “the part of the soul that craves recognition of dignity” (Fukuyama, 2018, p. 9). However, to a large part, personal identities are derived from social categories: people belong to various social groups, and the more people identify with those groups, the more they define themselves in terms of those groups. This is particularly important for developing self-worth and self-esteem. Being member of a highly evaluated social groups increases self-worth and self-esteem, while being member of a disrespected group threatens the personal identity.

The evaluation of social categories is defined by the recognition those categories experience from other people, institutions, or the society. Material or social incentives accruing from being a member of a group inform about the standing of the group in the recognition order of the society (Tajfel and Turner, 1986), or in other words, the treatment and evaluations members of certain groups receive from other groups determines the social recognition individuals perceive. In turn, social recognition is highly important for the identification with the social order or society as well as for the support of related institutions and processes (Honneth, 1995): while members of highly recognized groups will value the established social order, disrespected groups might challenge it. Previous research (e.g., Sanders et al., 2011) has reported social recognition as a significant predictor of political attitudes and behaviors. For instance, migrants show more positive evaluations of the host country when recognized (Huo and Molina, 2006; Just, 2017). Similarly, pro-LGBTQ people are more likely to participate in elections in LGBTQ-friendly states than in non-LGBTQ-friendly states (Ayoub and Page, 2020).

However, there are different types of good or bad evaluations people can receive for being member in certain categories. Fukuyama (2018) distinguishes two types of social recognition: “Isothymia” is the demand to be respected on an equal basis with other people; while “Megalothymia” is the desire to be recognized as superior (Fukuyama, 2018, p. 9). Not being respected as equal may pose a serious threat to a personal identity, as disrespect in this way lowers self-esteem considerably. But not being respected for a (self-perceived) superior capacity or achievement may also evoke bad feelings. Unfortunately, the Isothymic demand and Megalothymic demand may conflict since attempts to meet the demand for more equal recognition of one group will potentially diminish the privileged recognition of another group.

This brings us to the impact of inclusion politics on satisfaction with the democracy. Enhancing the recognition of social groups (or: fulfilling their demands for Isothymia), which is either a declared goal or a by-product of inclusive policies, would improve the evaluation of the personal identities of the members of those groups, and in turn, would improve their identification with and their support of the political system.

However, due to competition for social recognition between groups in a social hierarchy (Honneth, 1995), the improvements in recognition for the groups targeted by inclusive policies tend to go hand in hand with less privileges for other social groups, which might threaten their demands for Megalothymia. If, for example, migrant integration policies enhance the support of Isothymia of migrants, this could at the same time challenge the Megalothymia of natives, whose superior identity would be threatened by diminishing their privileges. Thus, political change toward more inclusive institutions also can foster identity threats among traditional privileged groups.

Empirical research already found some hints that point in that direction. For example, national nostalgia is found to be positively related to opposing more rights for migrants (Smeekes et al., 2015) who are perceived as bringing and practicing incompatible culture to the host country (Ketola and Nordensvard, 2018; Norris and Inglehart, 2019) and believed as undeserving of social citizenship rights and welfare benefits (Reeskens and van Oorschot, 2012). These resentments can further influence political attitudes and behavior of natives in ways opposing inclusive policies (Sanders et al., 2011).

Taken together, these recognition cleavages between social groups posited by Social Identity Theory motivate our central hypothesis (Honneth, 1995; Fukuyama, 2018): inclusive policies strengthen the Isothymia of disadvantaged social groups and in consequence, increase their democratic satisfaction. At the same time, they threaten the Megalothymia of privileged social groups and in turn, decrease their democratic satisfaction. This can either lead to a polarization in respect to democratic satisfaction, or to a convergence, depending on the initially observed level of democratic satisfaction of the respective advantaged and disadvantaged groups. If the democratic satisfaction of the advantaged group is initially higher compared to the satisfaction of the disadvantaged group, then inclusion politics for the disadvantaged group may lead to a convergence. But when the initial democratic satisfaction of the disadvantaged group is comparably higher or the two groups don't differ in their initial satisfaction level, then inclusive policies would lead to an increasing gap in democratic satisfaction between advantaged and disadvantaged groups indicating polarization.

### 2.2 Social groups, social inclusion, and political attitudes

Arguably, religion, migration, sexuality and gender issues became more prominent topics of political debate during the 1960s and 1970s (Inglehart, 1990; Wilson, 2006). Also, over the same period, many liberal democracies have had to rapidly adapt their social and economic institutional arrangements, resulting from macro-processes of social change such as globalization and modernization (Norris and Inglehart, 2019). Since then, policymakers are becoming increasingly aware that social, economic, and political exclusion promotes negative attitudes and behaviors toward the political system and associated democratic norms by excluded groups (Sanders et al., 2011). Thus, in many countries we can observe increasing attempts to establish integrative policies, targeting at different unequal rights and affecting cleavages for recognition between various social groups.

Against this background, we look at the effects of inclusive policies regarding: (1) religious freedom, (2) integration of migrants, (3) equal treatment of homosexuals, and (4) the promotion of gender equality on democratic satisfaction. We expect that these four inclusive policies affect five different cleavages about social recognition between isothymic and megalothymic social groups: Policies targeting religious freedom strengthen the Isothymia of non-religious and threaten the Megalothymia of religious people; migrant integration policies foster the Isothymia of migrants and undermine the Megalothymia of natives as well as strengthen the Isothymia of non-xenophobes and threaten the Megalothymia of xenophobes; policies on LBGTQ rights foster the Isothymia of non-homophobes and undermine the Megalothymia of homophobes; finally, gender equality policies strengthen the Isothymia of women and threaten the Megalothymia of men. In the following sub-sections, we present an overview of preceding studies on the effects of inclusive policies on the respective recognition cleavages.

#### 2.2.1 Religious freedom and religiosity

It is almost consensual among scholars that higher religiosity leads to more cohesive political attitudes like democratic satisfaction, institutional trust, and other support for conventional democracy (Lubbers et al., 2002; Norris, 2005; Werts et al., 2012; Montgomery and Winter, 2015; Cremer, 2023). What remains astonishing, however, is that conventional democratic institutions attract more support from religious people despite that anti-establishment politicians portray these institutions as the destroyer of traditional values (Lubbers et al., 2002; Norris, 2005; Werts et al., 2012; Montgomery and Winter, 2015; Cremer, 2023). That is, religious people still stand by the conventional democratic institutions who promote religious freedom allowing non-religious people to exercise their rights.

This paradox is explained by the “vaccine effect” theories that religious people have pre-existing identification with religious parties promoting empathy, solidarity and other values that are in contrary to the focus of anti-establishment parties (Arzheimer and Carter, 2009; Montgomery and Winter, 2015; Cremer, 2023). But it is not clear whether the cohesive relationship stays regardless of the level of religious freedom which may remove material and morally relevant privileges of the religious group.

Generally, religious communities oppose progressive reforms that come with religious freedom, especially rights related to sexual freedom and breaking up the traditional gender roles (e.g., Nicolet and Tresch, 2009; Adkins et al., 2013; Valenzi, 2022). Therefore, already Bloom and Arikan (2012) assert contrary to the “vaccine effect” that as the traditional religious values come under threat through religious freedom for all, less democratic support becomes more widespread among the religious group while the non-religious group show more support for democracy (Bloom and Arikan, 2012). Thus, the cleavage for recognition between religious and non-religious is steered up by policies promoting tolerance of a modern lifestyle (Nicolet and Tresch, 2009). In consequence, we expect that policies promoting religious freedom strengthen the Isothymia and democratic satisfaction of non-religious people while they threaten the Megalothymia of the religious group and decrease their democratic satisfaction.

Although there is much literature on the relationship between religiosity and political behavior, apparently no studies have been investigating the effects of varying degrees of religious freedom policies on the relationship between religiosity and political attitudes. We investigate how the “vaccine effects” (higher democratic satisfaction of religious compared to non-religious people) persist given increasing levels of religious freedom policies, which to our knowledge has not been done so far.

#### 2.2.2 Migrant integration, nativism, and xenophobia

The relationship between migration background and political attitudes has been extensively researched with most studies showing that migrants on average are more likely to show more democratic satisfaction, pro-EU integration parties voting, and other “cohesive” attitudes, than their native counterparts (Weldon, 2006; Wenzel, 2006; Maxwell, 2010; Röder and Mühlau, 2011, 2012; Sanders et al., 2011; Montgomery and Winter, 2015; Ketola and Nordensvard, 2018). This gap, which is explained by the “frame of reference effect” or reference-point hypothesis—i.e., lower expectations regarding political institutions and regulation of migrants from countries with poorer institutional performance—weakens over time with increased acculturation in the host country (Wenzel, 2006; Maxwell, 2010; Röder and Mühlau, 2011, 2012; Just, 2017).

However, how would migrants and natives react to increasing levels of migrant integration policies? Following the theories of recognition and identity politics explained earlier, favorable migrant integration policies would strengthen migrants Isothymia and make migrants show more democratic satisfaction. On the other hand, natives may not agree with strong migrant integration policies as they will remove their “special rights” and make them to compete equally with migrants in the social, economic, and political realms. Since nativism predicts worsening attitudes toward democratic institutions while migrants are more likely to identify with democratic institutions, gaps in democratic satisfaction between migrants and natives should become wider as migrant integration policies get stronger.

So far, studies have shown that positive migrant integration policies contribute to migrants' cohesive political attitudes and behavior (Huo and Molina, 2006; Sanders et al., 2011) while negative migrant integration policies reduce their democratic satisfaction and increase their quest for better recognition (Just, 2017). Nevertheless, literature on the relationship between migrant integration policies and natives' political attitudes remains inconclusive. We will add to this stream of research by using a wider database (see below) and most importantly show the effects of integration policy on both migrants' and natives' democratic satisfaction.

Looking deeper into migration concern, we argue further that migrant integration policies may not pose threats to all natives but only to a sub-group adhering to xenophobic attitudes. Apparently, most previous studies on migrant integration policies and political attitudes tend to focus on natives as a homogenous group (e.g., Mughan and Paxton, 2006; Weldon, 2006; Duina, 2020). Instead, we argue that there may be social polarization between those who see immigration as a threat to a nation, its' identity and cohesion and those who do not (hence referred to as “xenophobes” and “non-xenophobes”, respectively).

Some studies—following social identity threat theories—find increases in migrant integration policies as a factor instigating xenophobic attitudes (Bratton, 2002; Mughan and Paxton, 2006; Bartram and Jarochova, 2022). Additionally, where immigration policy is pro-immigrants, xenophobes are more likely to be politically distrustful (Mughan and Paxton, 2006; Solodoch, 2021) and even seek “alternatives” to conventional democratic institutions (Betz, 2009, p. 664; Just, 2017; Solodoch, 2021). Contrarily, a couple of studies—supporting intergroup contact theories—posit that migrant integration policies reduce xenophobic attitudes (e.g., Schlueter et al., 2013; Hooghe and de Vroome, 2015; Callens and Meuleman, 2017) especially when such policies promote assimilation and less economic benefits for migrants (Neureiter, 2022), while others conclude that no relationship exists at all between migrant integration policies and xenophobic attitudes (Meuleman and Reeskens, 2008; Schlueter et al., 2013; Hooghe and de Vroome, 2015; Bartram and Jarochova, 2022). Therefore, migrant integration and identity politics remain a contentious issue in public discourse and political research.

Arguably, this study contributes to this discussion not only because the relationship between migrant integration policy and xenophobic attitudes remains contentious, but also because it goes further to investigate how migrant integration policies may moderate the relationship between xenophobic attitudes and democratic satisfaction using a robust database. Thus, it sheds light on the context in which gaps in political attitudes between non-xenophobic and xenophobic people may shrink or get wider.

#### 2.2.3 LGBTQ rights and homophobia

Even more than gender, sexual rights are another dimension of social identity which is hotly debated in Europe. The recognition of sexual minorities is still being denied, especially in the Eastern part of Europe where homosexuality is viewed as a threat to the national identity as well as traditional moral values and has been used to legitimize homophobic rhetoric and behavior (Mole, 2011, 2016; Mole et al., 2021). Thus, regarding sexual minorities, we can observe a sharp cleavage between minorities together with their allies standing for Isothymia and some people with a high degree of Megalothymia, believing that their way of life can claim a decent moral superiority.

As such, efforts to promote LGBTQs as being equal in social recognition to heterosexuals might prompt sharp reactions of homosexual minorities and their empathisers on the one hand and homophobic people on the other hand. In general, we expect negative reactions from homophobes (Fejes, 2008; Tschantret, 2020) as their Megalothymia might be threatened by inclusionary policies regarding sexual freedom. Moreover, we expect positive reactions from homosexual minorities and their empathisers, as their Isothymia will be improved. A recent study presents first results pointing in that direction: non-homophobes are less likely to show political apathy in states promoting LGBTQ-friendly policies, compared to states discouraging LGBTQ-friendly policies (Ayoub and Page, 2020).

#### 2.2.4 Gender equality and patriarchy

Generally, men predominate among those with high political satisfaction (Anderson and Guillory, 1997; Sahin and Akboga, 2021) and supporters of right-wing and populist parties (Betz, 2009). In other words, their democratic satisfaction mirrors their privileged socio-economic position, and their party-preference show the willingness to maintain such existing power arrangements and counteract gender equality policies (Zagarri, 2007; Sanbonmatsu, 2008). On the other hand, as women are socially disadvantaged, they on average show less democratic satisfaction and more often support left-of-center political parties which favor equal opportunity policies (e.g., Iversen and Rosenbluth, 2006).

Given this pronounced initial gap in democratic satisfaction by gender, promoting gender equality by political means should increase the Isothymia and democratic satisfaction of women while it should threaten the Megalothymia of men and decrease their democratic satisfaction. In consequence, gender equality policies are expected to narrow the initial gap in democratic satisfaction between men and women.

#### 2.2.5 Summary and hypotheses

Theoretical considerations and empirical findings presented so far let us to expect that integrative policies support the demands for Isothymia of its “target groups”, resulting in an improvement of their democratic satisfaction, while it challenges the demands for Megalothymia of the corresponding “counter groups”, diminishing the democratic satisfaction of those groups. Taken together, this argumentation leads us to formulate the following set of hypotheses:

H1: Stronger inclusive policies improve the democratic satisfaction of non-religious groups, migrants, non-xenophobes, non-homophobes, and women, while the democratic satisfaction of religious groups, natives, xenophobes, homophobes, and men decreases.

As argued above and given the trends of H1, the characteristic of the overall pattern of group-specific democratic satisfaction and inclusive policies will in addition depend on the initial levels of democratic satisfaction of the groups under investigation. Based on the state of research on these group-specific levels of democratic satisfaction presented, two further hypotheses can be formulated:

H2a: As religious groups and men on average show a higher democratic satisfaction than non-religious people and women, stronger inclusive policies will narrow the differences in democratic satisfaction between these groups.
H2b: As migrants and non-xenophobes on average show a higher democratic satisfaction than natives and xenophobes, stronger inclusive policies will widen the differences in democratic satisfaction between these groups.

As we do not find previous results or convincing theoretical argument on differences in democratic satisfaction between homophobes and non-homophobes, we cannot derive a hypothesis on the influence of inclusive policies on these differences for this cleavage.

## 3 Data, research design, and methodology

### 3.1 Analytic strategy

For our analyses, we use multilevel models to explore how inclusive policies, which are measured at the country level (in the following also referred to as “level 2” units), affect the democratic satisfaction of the citizens, who constitute the “level 1” units of our models. We exploit data from the European Social Survey (ESS) rounds 1–10 [European Social Survey European Research Infrastructure (ESS ERIC), 2023], which contain all necessary information at level 1, and combine it with the relevant context information stemming from various sources. A common problem of this kind of multilevel analyses is the rather low number of observations at level 2. Thus, we try to use as many level 2 cases as possible for the various models. The main problem here is to find the needed context information. Measurements for the various indicators of inclusive policies are available only for a subset of the surveys in the ESS.

To overcome this problem, we apply the following strategy: Firstly, we use separate analysis samples for each of the five comparisons between the social groups. That means particularly that we use only one indicator of inclusive policies for each group comparison, avoiding additional missing information on level 2 and problems of multicollinearity between too many level 2 variables at the same time. Secondly, we mostly have multiple surveys of the included countries spanning a larger time frame available. Using several surveys per country conducted at different time points does not only increase the number of observations at level 2 but also allows us to separate between- and within-country effects which allow a step toward the causal interpretation of our results. Thirdly, where possible, we interpolate some measurements of our level 2 characteristics to match them with the country-specific observation windows of the ESS. To determine the time of the survey we use the year where an interview was conducted.

In total, we use data from 36 countries, spanning a time frame from 2002 to 2021. However, the single analysis uses only a subset of this data, resulting in different distributions for the variables used. Therefore, we show the distributions of the variables used for each analysis sample separately (see Supplementary Tables S6a–S10c).

### 3.2 Variables

The dependent variable is *democratic satisfaction* derived from the ESS item “how satisfied are you with the way democracy works in your country” (with 11-point scale from 0 = Extremely dissatisfied to 10 = Extremely satisfied).

The main independent variables on level 1 capture the social groups which are addressed by the various inclusive policies. Two of them are also demographic groups: *gender* and *migration background* (migrants—as any respondents who are either themselves born abroad or for whom one or both parents were born abroad, vs. natives—as respondents with both parents born in the country). As demographic characteristics they are included as controls in all our models, but only for the analyses of the inclusive policies where they are viewed as the main independent variables on level 1, we compute a cross-level interaction of these group indicators with the corresponding inclusion policy index on level 2.

Gender and migration background are ascribed characteristics based on institutional regulations. In this sense, they form “structurally” constructed groups which contribute to social identities and are subjected to social policies. By contrast, the groupings for the other three comparisons—religiosity, xenophobia and homophobia—are “culturally” constructed based on attitudes. On the one hand, that could mean that they do not contribute to social identity as rigidly as the ascribed characteristics, because attitudes can change and adapt to political measures. On the other hand, these components of identity reflect internalized worldviews which might contribute to social identity even more clearly than ascribed characteristics. For these attitude-based groupings, we contrast smaller groups with extreme views (e.g., not at all homophobic people vs. the very homophobes) and a larger group with mixed views. To identify homophobes, we use one item of the ESS (“gay men and lesbians should be free to live their own life as they wish”) where people who strongly agree are rated as non-homophobes, while we put the few people who disagree or strongly disagree into the group of homophobes. For religiosity and xenophobia, we first apply a factor analyses to three indicators for the respective latent construct (religiosity: “how religious do people rate themselves”, “how often do they attend to religious services”, “how often do they pray”; xenophobia: “immigration is good or bad for the country”, “cultural life is undermined or enriched by immigrants”, “immigrants make the country to a worse or better place”, see Supplementary Tables 11a, b) and estimate factor scores. Afterwards, we create three groups based on these scores, putting the lowest and highest scoring 20% into the respective “extreme” categories and the rest in the middle.

The shared level 1 control variables for all analyses are gender, migration background, age, years of education, employment status, the subjective financial situation and the political interest (both measured on a 4-point scale), the political self-positioning on the left-right-spectrum (measured on a 11-point scale), and party-closeness (1: people feel close to a particular party, vs. 0: not). For all metric variables, we exclude missing values by listwise deletion. We also exclude observations with missing information on migration status and gender as these are very few cases. However, for party closeness and, employment status, we kept cases with missing information in the analysis samples by including them in “other” category.

As indicators for the four inclusive policies investigated, we use the Migrant Integration Policy Index (MIPEX) (Solano and Huddleston, 2020), the Religious Freedom Index and Civil Liberties Index of the Global State of Democracy (Skaaning, 2020) and the Gender Equality Index (World Bank, 2022). The MIPEX's overall score is the average of eight migrant policy strands including labor market mobility, family reunification, education, political participation, permanent residence, citizenship, antidiscrimination, and health, from year 2007 to year 2019 with a few missing values interpolated purposely for this study. The religious freedom index (1975–2020) comprises of aggregated subcomponents including two general indicators on religious freedom based on expert surveys and two similarly broad in-house coded variables. The civil liberties index represents how well civil rights and liberties are respected in each country over some years with aggregated values from five civil liberties subcomponents including freedom of expression, freedom of association and assembly, freedom of religion, freedom of movement, and personal integrity and security. The gender equality index is based on assessments of the country-specific policies and institution regarding gender equality. Each of these policy indicators is only used for analyses of the respective cleavages between social groups. In consequence, only the MIPEX is used for two group comparisons: migrants vs. natives, and xenophobes vs. non-xenophobes.

However, in all models we control for the following country level economic and political characteristics: The GDP per capita and the Government Effectiveness Estimate extracted from the World Development Indicators (World Bank, 2022) and the Gini index (Gapminder, 2022). Investigating the differences in democratic satisfaction between migrants and natives, we additionally include the net migration rate, also using data from the World Development Indicators.

Before entering the analysis, all metric variables are z-standardized based on the respective analysis samples.

### 3.3 Methods

We employ multilevel mixed models, using lavaan v. 0.6.15 (Rosseel, 2012) of R v.4.3.2 (R Core Team, 2021). In general, we compute five models. The first model includes only the level 1 variables and a random intercept reflecting the country and time of the survey. Next, we include the respective inclusion policy index, a random coefficient for the social groups of interest, as well as a cross-level interaction between the group variable and the policy index, to assess the overall effect of the policies on democratic satisfaction and—most importantly for our study—how the strength of the policies affects the differences in democratic satisfaction between the respective groups. Here, we see the “gross” effect of the policies. Successively, we add the level 2 economic controls in model 3 (GDP and Gini index, and for the migrants vs. natives-analysis also the net migration rate) and the government effectiveness in model 4. Formally, the model we use in our step-wise analyses is given by:


(1) $$\begin{array}{l} y_{ij}=a_{j}\;+\;X_{ij}\;*\;\bar{b}\;+\;m\;*\;p_{j}\;+\;n_{j}\;*\;g_{ij}\;+\;o*\;p_{j}\;*\;g_{ij}\\ \;+\;Z_{j}\;*\;\bar{c}\;+\;e_{ij} \end{array}$$

with y_ij_ the democratic satisfaction of individual i in survey (i.e., country-year) j, a_j_ a survey specific random intercept, X_ij_ a matrix of individual-level explanatory variables, and $\bar{b}$ a vector of related fixed effects (Model 1). Model 2 adds the fixed effect m of the survey-level policy index of interest p_j_ (i.e., MIPEX), a random effect n_j_ for the contrasted social groups of interest g_ij_ (i.e., natives and migrants), and a fixed effect o for the interaction between policy index and group belonging. Model 3 and 4 add a matrix of survey-level explanatory variables Z_j_ and a vector of related fixed effects $\bar{c}$. e_ij_ is an individual-level error term (residual) included in all steps of the analyses.

Model 5 finally decomposes all the level2 variables in a between-country and a within-country (i.e., a time related) component and estimates a cross-level interaction with the respective social groups for each of the components of the policy index of interest. This allows us to assess how much of the variation in the effects of the focal variables is due to different levels of inclusive policies between countries and how much is due to changes over time within countries. The latter is especially interesting as it removes—analogous to a fixed effect model—unobserved heterogeneity between countries, moving a step toward causal inference (Fairbrother, 2014).

For the analysis of the effects of the MIPEX indicator on differences in democratic satisfaction between migrants and natives, an inspection revealed a non-linear relationship between these variables. Therefore, we use an additional quadratic term for the MIPEX in the respective models.

## 4 Results

In the following, we present our analysis investigating the impact of inclusive policies on the differences in democratic satisfaction between social groups. As outlined above, we look at four policies affecting five group contrasts. We did a separate analysis for each group comparison, focusing on the influence of the respective integration policy.

In all the models we control for some variables that could covary with the characteristics of our social groups compared and thus, lead to differences in democratic satisfaction which are not due to unequal social recognition of group identities. Most of these variables show the effects on democratic satisfaction which are known from previous studies [see Model 1 (M1) in Supplementary Tables S1a, S2a, S3a, S4a, S5a]: males, people with a migration background, younger, higher educated, and people having a full-time job show a higher degree of democratic satisfaction. People with a higher political interest and a higher subjective economic status are more satisfied with democracy. Also, people who feel close to a certain party show a higher degree of satisfaction, as well as more rightist respondents.

Furthermore, for the level 2 control variables [see Model 4 (M4) in Supplementary Tables S1a, S2a, S3a, S4a, S5a] which could covary with the inclusion policies investigated we find that in countries with a higher GDP and a higher government effectiveness, people are on average more satisfied with democracy, while in countries with a higher degree of economic inequality indicated by Gini the average democratic satisfaction is lower.

**Figure 1:**
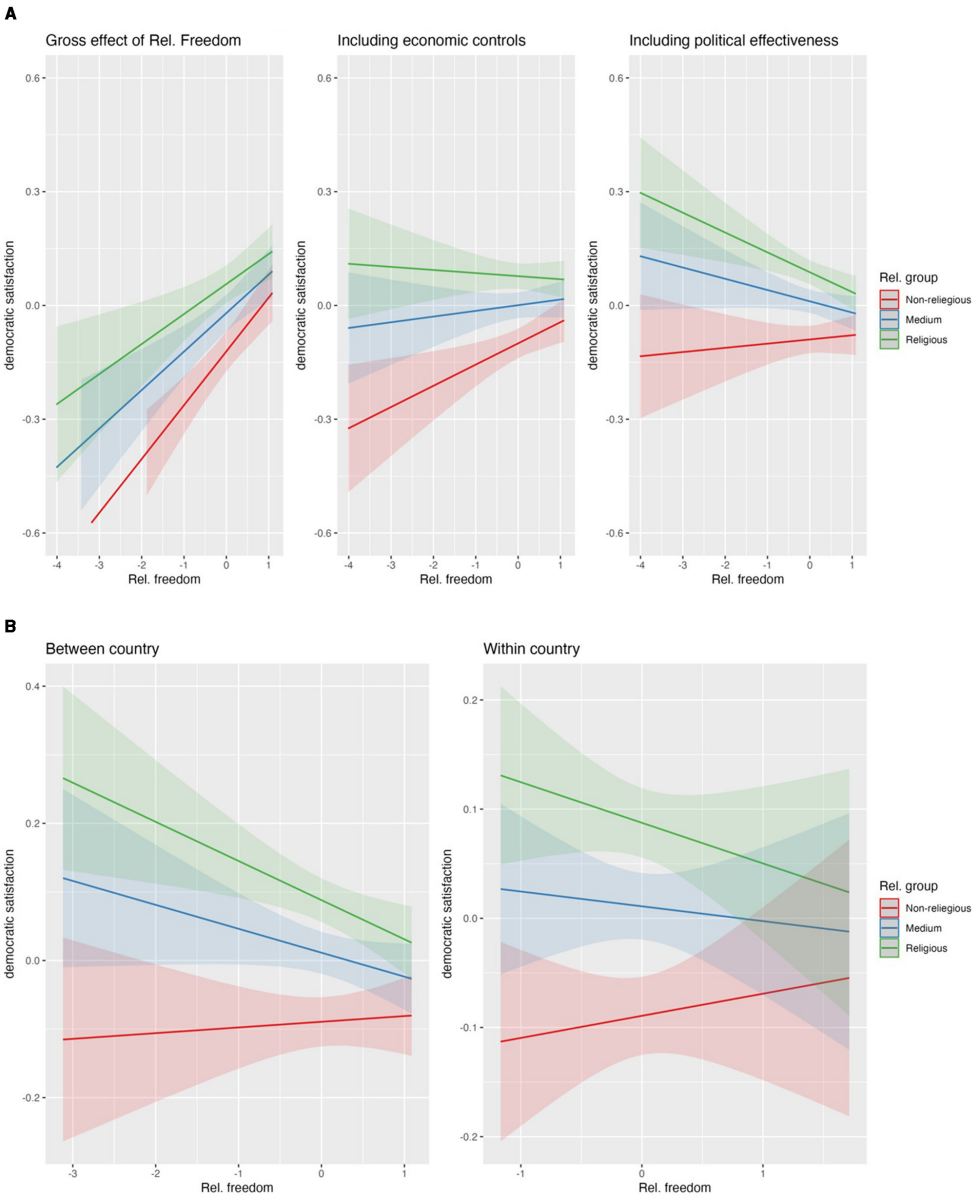
**(A)** Impact of religious freedom on religious-non-religious gap. **(B)** Impact of religious freedom on religious-non-religious gap, separating country-time effects.

**Figure 2:**
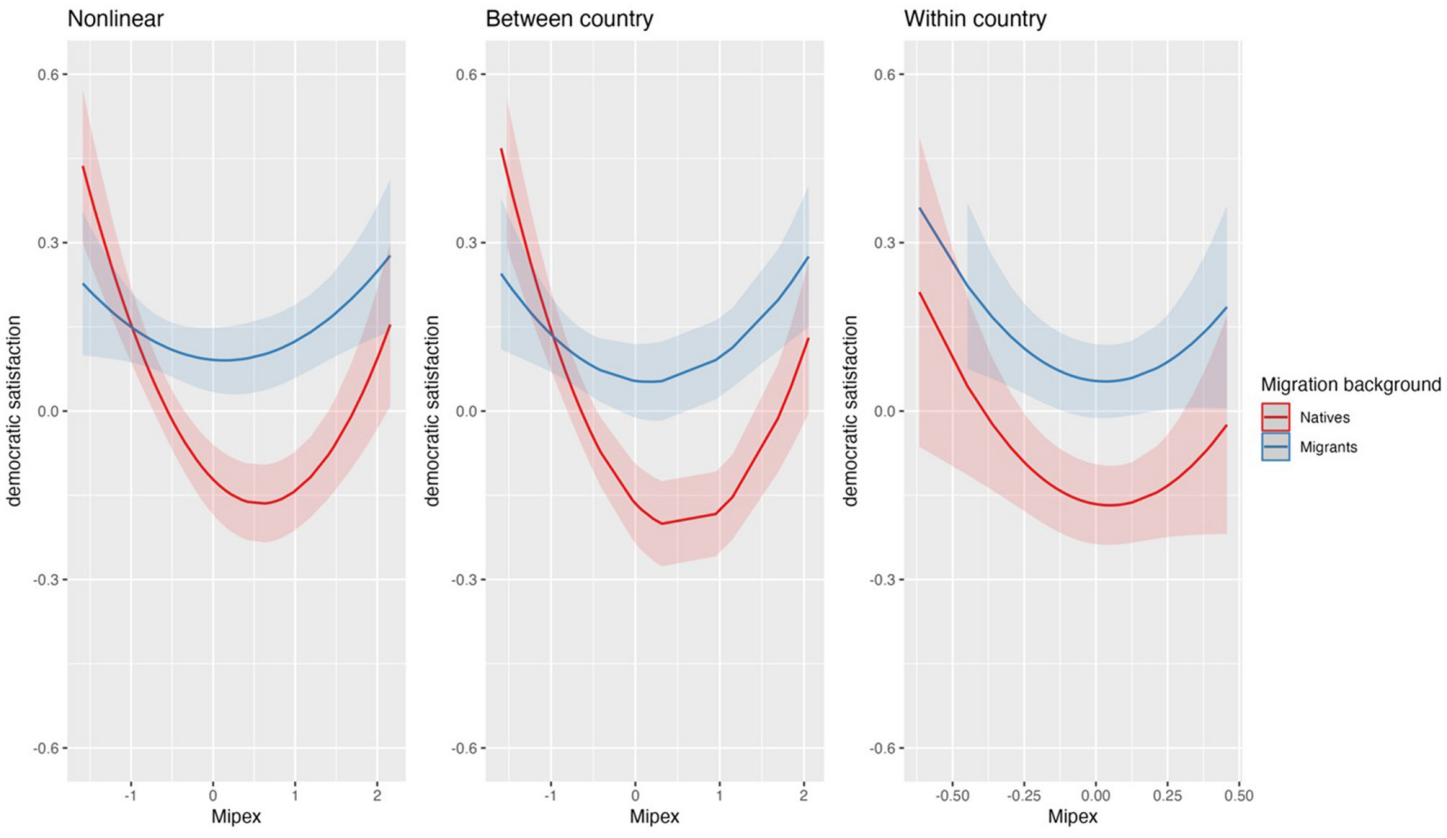
Impact of MIPEX on migrants-natives gap, non-liner.

**Figure 3:**
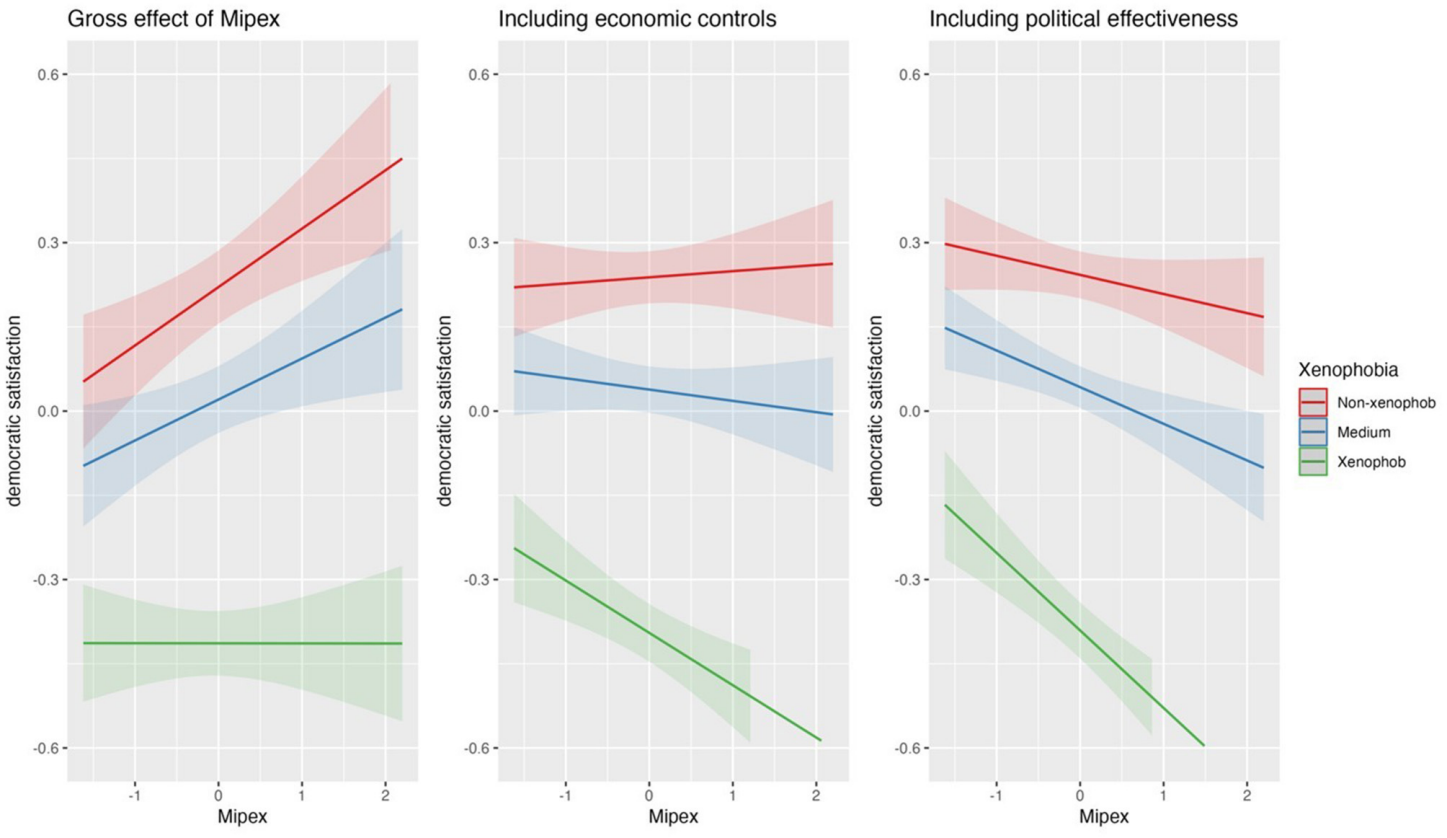
Impact of MIPEX on xenophobes-non xenophobes gap.

**Figure 4:**
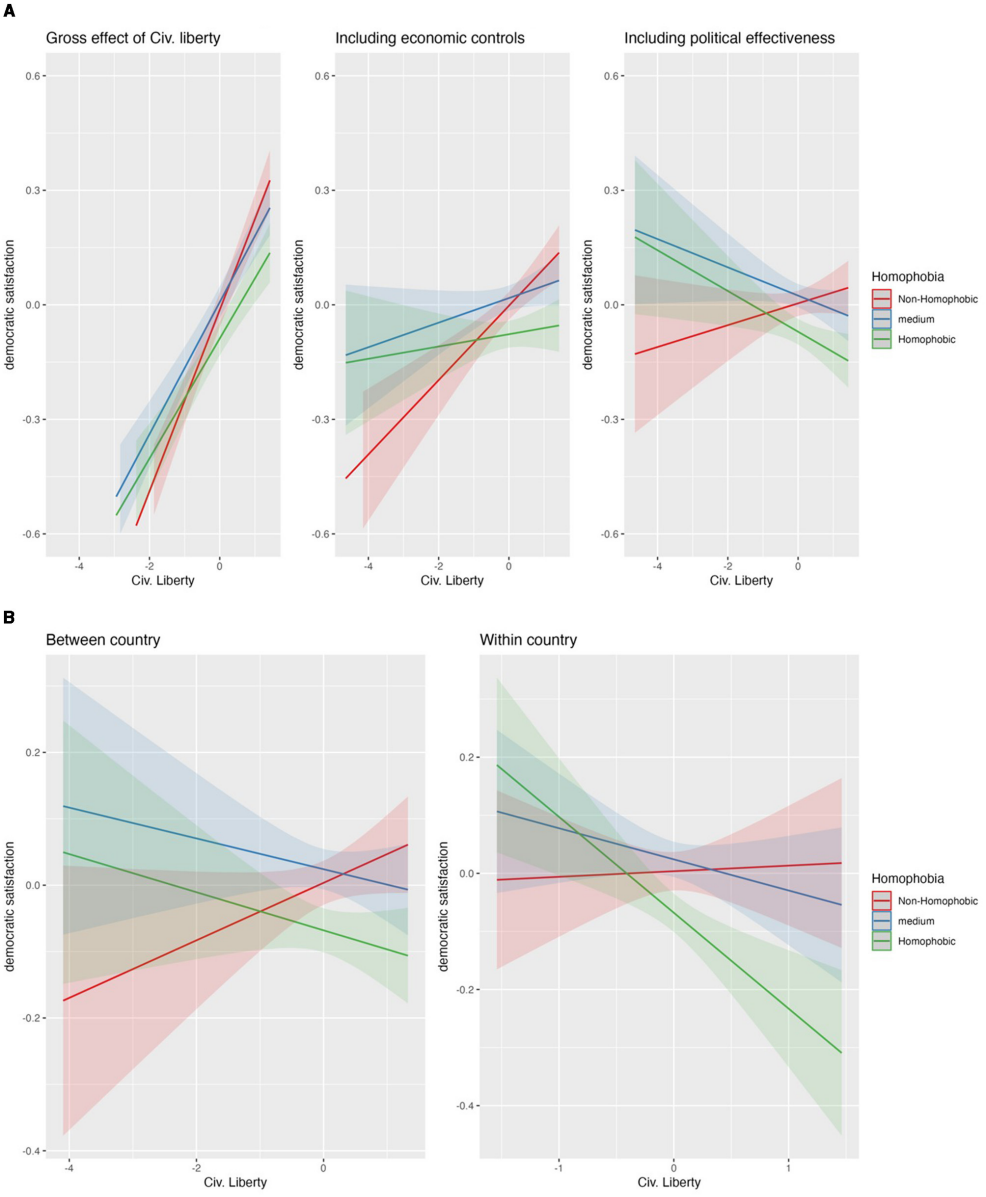
**(A)** Impact of civ. lib. index on homophobes—non homophobes gap. **(B)** Impact of civ. lib. index on homophobes—non homophobes gap, separating country-time effect.

**Figure 5:**
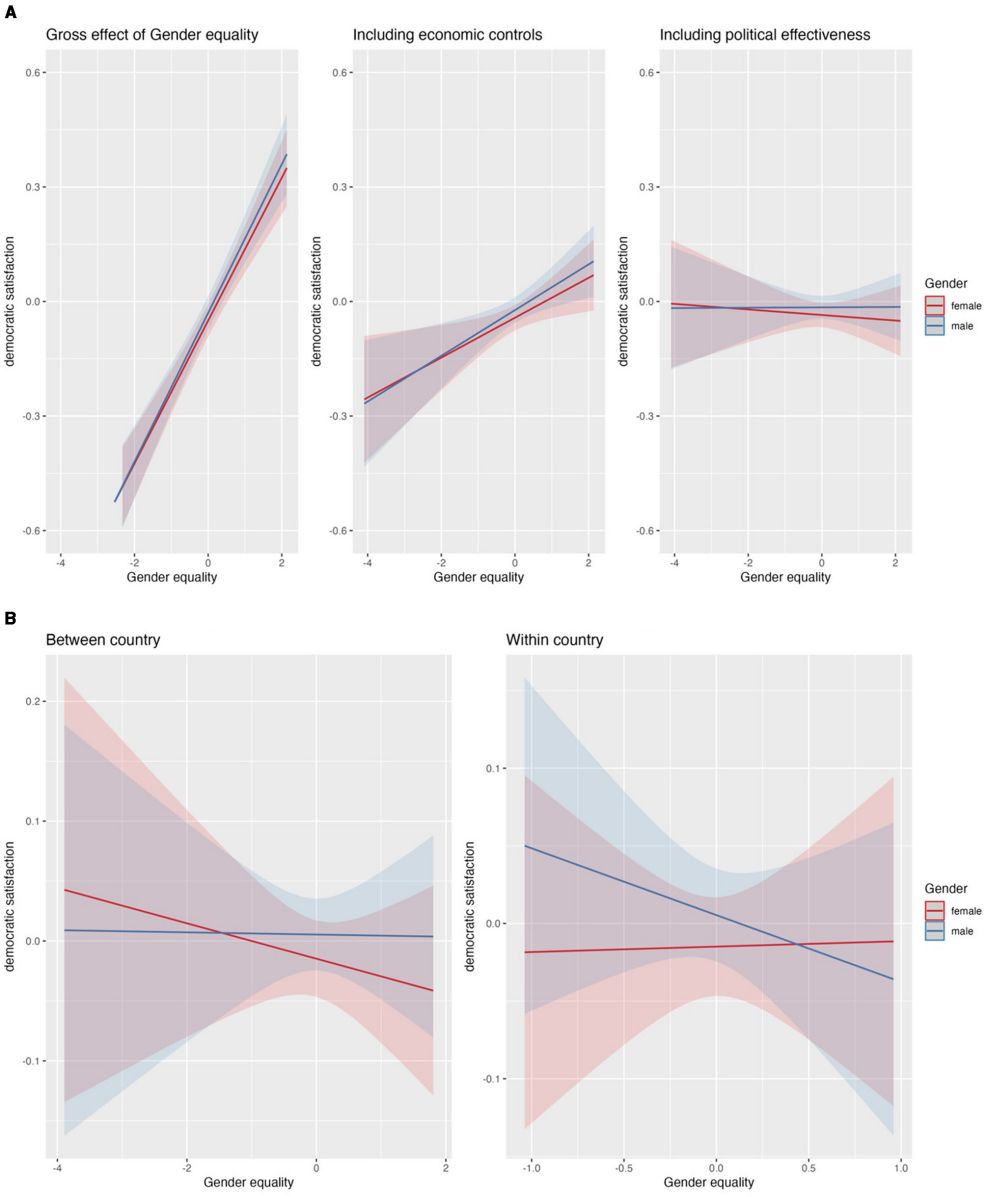
**(A)** Impact of gender equality index on gender gap. **(B)** Impact of gender equality index on gender gap, separating counry-time effects.

Next up, we discuss the results for the different social group and inclusion policy indicators in more detail. The related result tables are included in see Supplementary Tables S1a–S5c while additional graphs of the central results are shown in the main text (see Figures 1A–5B).

### 4.1 Religiosity and the religious freedom index

Each of our group comparisons starts with a model that includes only the level 1 variables to estimate the average differences between the target groups in respect to their democratic satisfaction, adjusted for possible confounders at the individual level (M1). In Supplementary Table S1a M1 we see that higher religiosity tends on average to increase democratic satisfaction. According to H1, we would expect the democratic satisfaction of religious people to decrease with policies supporting religious freedom while the satisfaction of non-religious people increases. Given the initial levels of democratic satisfaction of these groups, H2a leads us to expect these group differences in democratic satisfaction to shrink with stronger religious freedom policies.

Including the cross-level interaction with the degree of religious freedom granted in a society (Supplementary Table S1a, M2), we see that in line with H2a the differences in democratic satisfaction between the religious groups indeed are lower when religious freedom is larger. However, Figure 1A (first panel) shows no support for H1: for religious and non-religious people we can observe that their democratic satisfaction increases when the degree of religious freedom of their country increases, but the slope being steeper for non-religious people.

Including the level 2 controls of the economic situation of the countries (GDP and Gini index, M3 in Supplementary Table S1a, panel 2 in Figure 1) and the effectiveness of the government (M4 in Supplementary Table S1a, panel 3 in Figure 1) a different picture emerges. Religious freedom is more predominant in wealthier countries with more effective governments, and all people are obviously more satisfied in those countries, irrespective of their religiosity. After adding the controls, the pattern expected in H1 emerges: the democratic satisfaction of the non-religious people *net of economic prosperity and effective governance* is higher in countries with a higher degree of religious freedom, while the democratic satisfaction of the religious groups is lower.

While both cross level-interaction effects are significant, emphasizing the “convergence” of the groups in respect to democratic satisfaction, only for the most religious group we see a significant association of their level of democratic satisfaction with the degree of religious freedom (see Supplementary Table S1c). That means, religious freedom does not influence so much the democratic satisfaction of the non-religious groups by fulfilling their demands for Isothymia, but it affects considerably the democratic satisfaction of the most religious group by challenging their demands for Megalothymia.

Separating between-country and within-country effects of religious freedom on democratic satisfaction (see Supplementary Table S1b, Figure 1B) we see strong group differences for the between-country component. Furthermore, the within country effect for the most religious group on democratic satisfaction is significant, too (Supplementary Table S1b) However, only the between-country association between the level of democratic satisfaction of the most religious group with religious freedom is significant (see Supplementary Table S1c).

In summary, our results are in line with previous studies as we can see a “vaccine effect” (Arzheimer and Carter, 2009; Montgomery and Winter, 2015; Cremer, 2023) on the one hand, and at the same time religious people opposing religious freedom policies (Nicolet and Tresch, 2009; Bloom and Arikan, 2012; Adkins et al., 2013; Valenzi, 2022). The granting of religious freedom challenges the Megalothymia of the religious people, decreasing their democratic satisfaction considerably, and improves the Isothymia of non-religious people, buffering their democratic satisfaction. Our study contributes to the existing knowledge by showing that the resulting impact on democratic satisfaction is a function of the degree of religious freedom and the initial discrepancy in support for democratic institutions between religious and non-religious people. This “vaccine effect” tends to fade out as policies supporting religious freedom get stronger.

### 4.2 Migration background, xenophobia, and MIPEX

In Supplementary Table S2a, we can see that migrants are on average more democratically satisfied than natives (M2). This result is in line with the “frame of reference effect”. Including the cross-level-interaction between migration background and MIPEX and successively adding the economic and political controls (M2-4) results in a similar pattern and supports H1: the more institutional regulations integrate migrants, the higher the democratic satisfaction of the migrants, but the lower the democratic satisfaction of the natives. However, inspecting the relationships between the variables revealed a more complex pattern as we need to include a quadratic term of MIPEX (see Supplementary Table S2b). Panel 1 in Figure 2 visualizes the resulting pattern: as MIPEX increases, the democratic satisfaction of the natives shrinks as expected—but only up to a certain level of integration. When countries reach a higher level of integration policies (about 0.5 standard deviations above average), the democratic satisfaction of the natives rises again. Surprisingly, the same relationship holds for the people with migration background, though the curve is much steeper for the natives. Only as MIPEX reaches around 1.0 standard deviations below average, the democratic satisfaction of migrants improves continuously. This more complex relationship between MIPEX and the democratic satisfaction of the two groups implies that in countries with a low level of integration policies natives are more satisfied with democracy than migrants, but this gap turns around when integration policies improve. Thus, given weak inclusive policies for migrants we find no “frame of reference effect” and only up to an average level of inclusive policies our results support H2b. Furthermore, our analyses show that the relationship between MIPEX and the democratic satisfaction of our focal groups is completely driven by the between-country component as there are no significant within-country effects (see Supplementary Table S2c).

Taken together, the non-linear relationship between MIPEX and democratic satisfaction of natives is plausible: the threat by migrants in general and by migrant integration in particular is especially large when the phenomenon of migration (and migration policies) starts to evolve. After a phase of acculturation, the natives seem to feel less threatened. This interpretation is in line with the “contact-hypothesis” as migrant integration policies might improve contacts between migrants and natives, at least for the more advanced countries in this respect.

Our finding that migrants are on average more democratically satisfied than natives is in line with previous findings (Weldon, 2006; Wenzel, 2006; Maxwell, 2010; Röder and Mühlau, 2011, 2012; Sanders et al., 2011; Montgomery and Winter, 2015; Ketola and Nordensvard, 2018). However, we refine this finding in so far as it is not the case given very weak migrant integration policies. Moreover, moving beyond the general conclusions of most studies that increase in migrant integration policies cushion the existing worries of the natives (e.g., Schlueter et al., 2013; Hooghe and de Vroome, 2015; Callens and Meuleman, 2017; Neureiter, 2022) or at least do not threaten the natives (Meuleman and Reeskens, 2008; Schlueter et al., 2013; Hooghe and de Vroome, 2015; Bartram and Jarochova, 2022) our findings shed more light: the integration policies appear threatening to the natives until the policies gets to its average but the policies improve the natives' satisfaction only where the policies are above average. The mechanism here may be that of anticipated threats at the beginning of more pronounced immigration processes and with the introduction of such policies which get weaker after acculturation with the new situation and with likely realized benefits given stronger integration policies.

When we focus on those people who have the most negative attitudes toward migrants—the Xenophobes—we get a different picture. Before controlling for the economic situation of the countries and government effectiveness, an increasing MIPEX increases the democratic satisfaction of the non-xenophobes but does not affect the satisfaction of the Xenophobes (see Supplementary Table S3a, Figure 3, first panel). However, after controlling for those level 2 characteristics, we see that an increasing MIPEX does not significantly affect the democratic satisfaction of the non-xenophobes but strongly decreases the democratic satisfaction of the xenophobes (see Figure 3, panels 2-3). These findings are in line with H1 for the xenophobes, but not for the non-xenophobe people. Furthermore, supporting H2b, we see a clear “polarizing” pattern: xenophobes even at low MIPEX levels show a much lower democratic satisfaction than the non-xenophobes and with stronger migrant integration policies these group differences in democratic satisfaction get larger which is also shown by the highly significant cross-level effects. Though the patterns are similar comparing the effects of between- and within-country components of MIPEX on democratic satisfaction, only the former show significantly increasing differences in satisfaction between the groups (see Supplementary Tables S3b, c).

Just like previous studies (Bratton, 2002; Mughan and Paxton, 2006; Betz, 2009; Just, 2017; Solodoch, 2021; Bartram and Jarochova, 2022) following social identity theories, we found that stronger migrant integration policies threaten xenophobes who in turn show less support for given democratic institutions. Thus, our results do neither support the “contact hypothesis” for xenophobes (e.g., Schlueter et al., 2013; Callens and Meuleman, 2017; Hooghe and de Vroome, 2015; Neureiter, 2022) positing that migrant integration policies reduce xenophobic attitudes, nor the conclusion that no relationship exists at all between migrant integration policies and xenophobic attitudes (Meuleman and Reeskens, 2008; Schlueter et al., 2013; Hooghe and de Vroome, 2015; Bartram and Jarochova, 2022). Overall, our findings suggest that xenophobes are not willing to give up their megalothymic demands.

### 4.3 Homophobia and the civil liberties index

In Supplementary Table S4a (M1), we see that homophobic people are on average less satisfied with democracy than non-homophobes. The significant cross-level-interactions (Supplementary Table S4a, M2) show that this gap narrows significantly when countries offer more Civil liberties. Like the analyses for religious freedom, we observe a strong improvement of democratic satisfaction for all groups with more civil liberties before controlling for the economic situation of the countries (see Figure 4A, panel 1). After controlling for economic and political country-characteristics the democratic satisfaction of non-homophobic people increases with increasing civil liberties, while the democratic satisfaction for medium and highly homophobic people shrinks (Figure 4A, panel 3). However, only the impact of increasing civil liberties on the democratic satisfaction of the most homophobic group is significant in line with H1 (see Supplementary Table S4c). Thus, we see that “too much” civil liberties threaten the megalothymic demands of the most homophobic group. Again, this pattern is like the analyses of religious freedom. However, we notice that the distribution of the civil liberties index is quite left skewed so that the big gaps at the long stretched lower tail of the distribution relies on comparably few observations. Possibly also for this reason, we see no clear convergence pattern as in the case of the analyses for religious freedom.

In addition, we find similar patterns for the effects of within- and between-country components of civil liberties on democratic satisfaction (see Figure 4B). Both the between- and the within-country components have significant interactions with the focal groups (see Supplementary Table S4b). However, only the impact of the within-country component of civil liberties on the democratic satisfaction of the most homophobic group is significant (see Supplementary Table S4c) which is a strong hint that an increase of civil liberties over time threatens the megalothymic demands of homophobic groups.

Our findings are in line with the conclusion of Ayoub and Page (2020) that non-homophobes, compared to homophobes, are more likely to support democracy in states promoting LGBTQ-friendly policies.

### 4.4 Gender and the gender equality index

Supplementary Table S5a (M1) provides evidence that men are more democratically satisfied than women. However, gender equality policies appear not to affect the gap in democratic satisfaction between men and women. The discrepancy between men and women in respect to democratic satisfaction remains stable as the government promotes gender equality and—like the analyses for religious freedom and civil liberties—before controlling for the economic situation of the countries democratic satisfaction strongly increases with stronger gender equality policies (see Figure 5A, panel 1). After controlling for economic and political characteristics of the countries at level 2, higher levels of gender equality do not affect the democratic satisfaction of men and women at all (see Supplementary Table S5a, Figure 5A, panel 2-3).

Distinguishing the effects of the between- and within-country components of gender equality policies on democratic satisfaction also shows no convincing related differences between men and women (see Figure 5B). While the respective gender related cross-level interactions are significant (see Supplementary Table S5b) hinting to contrary impacts for men and women, the overall associations of gender equality policies and the democratic satisfaction of the both genders are not (see Supplementary Table S5c).

Just as previous studies (Anderson and Guillory, 1997; Sahin and Akboga, 2021), this study finds men to be more democratically satisfied than women. However, gender equality policies appear not to affect the gap in democratic satisfaction between men and women. In sum, our findings suggest that gender equality policy does not pose a threat to the males as a whole group.

### 4.5 Robustness checks

To back up our analyses, we computed some additional models. Firstly, we lagged the variables at level 2, to grant some time to the contextual factors to unfold their impact. A lag of 1 year as well as a lag of 2 years did not change the results noticeably. Secondly, regarding our attitude-based group comparisons we also computed models using the continuous factor scores resulting from our factor analyses instead of constructing groups based on these scores, investigating how the marginal effect of a change in religiosity, xenophobia and homophobia change with increasing levels of integration policies. Those analyses lead to the same conclusions. Thirdly, we extend our models by adding controls for additional political characteristics of the countries (also based on the World Development Indicators): measures for political stability, voice and accountability, and control of corruption. As expected, including those indicators lead to problems of multicollinearity, especially with the indicator for government effectiveness, affecting the significance tests of the level 2 parameters. However, the patterns of the reported relationships are very similar.

## 5 Discussion

### 5.1 Summary

In this paper, we investigated how inclusive policies affect the democratic satisfaction of citizens. While there are quite a lot of studies doing research on this topic, especially in respect to the question how much disadvantaged groups gain from such policies, we focused on how these policies affect differences in democratic satisfaction between the disadvantaged groups which are the targets of those policies, and the corresponding privileged groups. We expected that we would repeat the results of the previous studies—which mostly found that disadvantaged groups improve in their democratic satisfaction—because inclusive policies are a signal for social recognition, fulfilling the demands for Isothymia of these group which would foster their self-worth and the support they perceive by societal institutions, enhancing in turn the satisfaction with these institutions. In contrast—and in addition to the pervious literature—we assumed that inclusive policies might threat the identities of privileged groups by diminishing their privileges and denying their demands for Megalothymia, which would in turn decrease their satisfaction with democratic institutions. Depending on the initial differences in democratic satisfaction between the compared groups, these effects of inclusive policies could result in either convergence or polarization in democratic satisfaction of the groups.

We looked at four different policies: increasing religious freedom, integration of migrants, increasing civil liberties and equalizing rights for women. We expected that increasing religious freedom would be a threat for the religious people, thus decreasing their democratic satisfaction, while the non-religious people would gain. Furthermore, migrant integration policies would affect the democratic satisfaction of people with migration background (fulfilling their demands for Isothymia) and xenophobic people (threatening their demands for Megalothymia). While increasing civil liberties would increase Isothymia for sexual minorities and their allies, they could threaten the Megalothymia for homophobic groups. And finally, Equalizing rights for gender would threaten men's identity while improving the democratic satisfaction for women.

Overall, the results of our study support those considerations quite well. Only for gender inclusive policies we did hardly find any impact on the democratic satisfaction of both genders: men are slightly more satisfied than women and this difference does not change with the degree of gender equality. For migrant integration policies, we found a non-linear relationship between differences in democratic satisfaction of migrants and natives with MIPEX: both groups show a lowering democratic satisfaction when the level of migrant integration policies changes from very low to medium, but satisfaction increases more pronounced with further increases of MIPEX. While this “initial drop” is somewhat surprising for migrants, this pattern is quite plausible for natives: increasing migrant integration seems indeed to threaten their demands for Megalothymia, but they get acculturated once the strength of the policies reach a certain level. In sum, our results show a “first polarization, then convergence” pattern with respect to the effects of migration integration policies on democratic satisfaction.

The remaining three cleavages and their association with inclusion policies is clearer in line with our hypotheses: in respect to religious, homophobic and xenophobic groups, we find that their democratic satisfaction shrinks when policies grant more rights to the corresponding counter-groups. Correspondingly, the democratic satisfaction of these counter-groups which are supported by these policies increases. However, somewhat surprisingly, and partly in contrast to previous findings, the gains in demographic satisfaction of the groups supported by inclusion policies is not large. After controlling for the economic conditions and the government effectiveness of the societies under study, the increases in democratic satisfaction are only minor and partly not significant anymore. Thus, the inclusion policies under study here correlate considerably with a general economic and political development of societies of which all people profit. After controlling for the economic and political conditions on the country level, the democratic satisfaction of the disadvantaged groups under study does not improve much from the related inclusive policies, while the threats for the more conservative counter-groups—declining the democratic satisfaction of those groups—become more apparent.

### 5.2 Conclusion

What does that mean for policy makers—should they avoid more liberal/ integrative politics to satisfy the Megalothymia of the more extreme conservative groups, as some politicians propose recently? Certainly not. The trends revealed in this study in most cases (migrants vs. natives, non-religious vs. religious, and non-homophobes vs. homophobes) lead to a convergent pattern of democratic satisfaction, leveling out the discrepancies between the groups in question. Only for Xenophobia we find a polarizing pattern but driven mostly by a small group of people. Also, the cleavages we have highlighted and studied are exactly those on which the far-right populists are currently gaining support in many countries. Through mass media, most especially social media, they promise to restore the traditional social recognition order for their supporters. In consequence, related right-wing populist parties gain votes through communication campaigns that focus on polarization and simplification. Specifically, those communication campaigns leverage contrasts between natives and immigrants, secular and traditional religious values, the role of men and women in the society, and the rights of hetero- and homosexual persons. Therefore, politicians are well advised to be aware of the threats that go along with inclusive policies and should communicate the benefits carefully to avoid challenging prevailing Megalothymia demands.

### 5.3 Limitations

For our analyses, we use a high-quality dataset. We test our theoretical idea by using very different groups assuming similar social mechanisms and come to very similar results. We used quite a bunch of robustness checks that corroborate the results. However, there are some limitations which should be addressed in further research.

First, we use observational data which is subjected to unobserved heterogeneity. Only for one of the four analyzed policies—the case of religious freedom, we find robust within-country (over time) effects which do not suffer as much from this problem. For the other policies there might be some unobserved heterogeneities between the countries which are responsible for the results we obtained. Within-countries (over time) differences in policies are usually far smaller than between-country differences, so incorporating more country characteristics in those analyses could be helpful. However, due to associated multicollinearity problems (see Section 4.5) this is contingent on surveys containing even more country-year observations than the current version of the ESS. Also, an experimental design like a vignette study could be used to allow for causal inference on the effects of perceived inclusion policies on democratic satisfaction.

Second, democratic satisfaction is only one possible outcome of strengthening or threatening social identities because of policies. Generalized trust, institutional trust and other political attitudes like populism could be investigated as well. Third, there are more social categories which can become targets of political action like generation or ethnic groups, with comparable impacts on political attitudes and behavior. Overall, the research on the consequences of certain policies for social cohesion by affecting social identities is only at its starting point.

## Data Availability

Publicly available datasets were analyzed in this study. This data can be found here: https://ess.sikt.no/en/series/321b06ad-1b98-4b7d-93ad-ca8a24e8788a.
