# Supplementary material for: Social inclusion policy effects on democratic satisfaction in Europe: a catalyst of polarization threating the identities of privileged social groups
Source: Front Sociol. 2025 Jun 4;10:1567394. doi: 10.3389/fsoc.2025.1567394 (PMC12174112; doi:10.3389/fsoc.2025.1567394)
Supplement: Supplementary file 1 [file Data_Sheet_1.pdf]

Table 1a: Impact of Religious Freedom on religious-non-religious gap

	M1	M2	M3	M4	L2 lagged 1Y	L2 lagged 2Y
(Intercept)	-0.122***	-0.128***	-0.110***	-0.100***	-0.101***	-0.100***
	(0.028)	(0.027)	(0.020)	(0.018)	(0.019)	(0.019)
Rel. group_Medium	0.099***	0.101***	0.101***	0.101***	0.100***	0.100***
	(0.009)	(0.009)	(0.009)	(0.009)	(0.009)	(0.009)
Rel. group_Religious	0.175***	0.178***	0.177***	0.177***	0.177***	0.177***
	(0.014)	(0.014)	(0.014)	(0.014)	(0.014)	(0.014)
Gender_male	0.039***	0.039***	0.039***	0.039***	0.039***	0.039***
	(0.003)	(0.003)	(0.003)	(0.003)	(0.003)	(0.003)
Mig. back._Migrants	0.118***	0.118***	0.118***	0.118***	0.118***	0.118***
	(0.004)	(0.004)	(0.004)	(0.004)	(0.004)	(0.004)
Age	-0.054***	-0.054***	-0.054***	-0.054***	-0.054***	-0.054***
	(0.002)	(0.002)	(0.002)	(0.002)	(0.002)	(0.002)
Education	0.019***	0.019***	0.019***	0.019***	0.019***	0.019***
	(0.002)	(0.002)	(0.002)	(0.002)	(0.002)	(0.002)
Activity_parttime	-0.019**	-0.019**	-0.020**	-0.020**	-0.020**	-0.020**
	(0.007)	(0.007)	(0.007)	(0.007)	(0.007)	(0.007)
Activity_self employed	-0.039***	-0.038***	-0.038***	-0.038***	-0.038***	-0.038***
	(0.006)	(0.006)	(0.006)	(0.006)	(0.006)	(0.006)
Activity_unemployed	-0.054***	-0.054***	-0.054***	-0.054***	-0.054***	-0.054***
	(0.007)	(0.007)	(0.007)	(0.007)	(0.007)	(0.007)
Activity_other	0.052***	0.052***	0.052***	0.052***	0.052***	0.052***
	(0.003)	(0.003)	(0.003)	(0.003)	(0.003)	(0.003)
Pol. int.	-0.053***	-0.053***	-0.053***	-0.053***	-0.053***	-0.053***
	(0.002)	(0.002)	(0.002)	(0.002)	(0.002)	(0.002)
Sub. hhincome	-0.143***	-0.143***	-0.143***	-0.143***	-0.143***	-0.143***
	(0.002)	(0.002)	(0.002)	(0.002)	(0.002)	(0.002)
Left-right	0.097***	0.097***	0.097***	0.097***	0.097***	0.097***
	(0.002)	(0.002)	(0.002)	(0.002)	(0.002)	(0.002)
Party close_no close party	-0.117***	-0.117***	-0.117***	-0.117***	-0.117***	-0.117***
	(0.003)	(0.003)	(0.003)	(0.003)	(0.003)	(0.003)
Party close_nk	-0.062***	-0.062***	-0.062***	-0.062***	-0.062***	-0.062***
	(0.012)	(0.012)	(0.012)	(0.012)	(0.012)	(0.012)
Rel. freedom		0.142***	0.056**	0.011	0.017	0.015
		(0.027)	(0.021)	(0.020)	(0.020)	(0.020)
Rel. group_Medium:Rel. freedom		-0.040***	-0.041***	-0.041***	-0.041***	-0.039***
		(0.009)	(0.009)	(0.009)	(0.009)	(0.009)

	M1	M2	M3	M4	L2 lagged 1Y	L2 lagged 2Y
Rel. group_Religious:Rel. freedom		-0.063***	-0.064***	-0.063***	-0.062***	-0.061***
		(0.014)	(0.014)	(0.014)	(0.014)	(0.014)
Gini Index			-0.057***	-0.041**	-0.040*	-0.044**
			(0.017)	(0.016)	(0.016)	(0.016)
Gdp			0.265***	0.139***	0.133***	0.135***
			(0.017)	(0.023)	(0.023)	(0.024)
Pol. effectivity				0.181***	0.184***	0.174***
				(0.025)	(0.026)	(0.027)
SD (Intercept group)	0.433	0.409	0.298	0.273	0.275	0.280
SD (Rel. group_Medium group)	0.129	0.124	0.123	0.123	0.123	0.124
SD (Rel. group_Religious group)	0.199	0.190	0.190	0.190	0.191	0.191
Cor (Intercept~Rel. group_Medium group)	-0.459	-0.391	-0.479	-0.514	-0.520	-0.520
Cor (Intercept~Rel. group_Religious group)	-0.418	-0.349	-0.528	-0.521	-0.520	-0.526
Cor (Rel. group_Medium~Rel. group_Religious group)	0.890	0.879	0.881	0.879	0.879	0.880
SD (Observations)	0.871	0.871	0.871	0.871	0.871	0.871
Num.Obs.	362520	362520	362520	362520	362520	362520
ICC	0.2	0.2	0.1	0.1	0.1	0.1

- $p < 0.1$, * $p < 0.05$, ** $p < 0.01$, *** $p < 0.001$

Table 1b: Impact of Religious Freedom on religious-non-religious gap,separating country-time effects

(Intercept)	-0.100***
	(0.018)
Rel. group_Medium	0.101***
	(0.009)
Rel. group_Religious	0.177***
	(0.014)
Gender_male	0.039***
	(0.003)
Mig. back._Migrants	0.118***
	(0.004)
Age	-0.054***
	(0.002)
Education	0.019***
	(0.002)
Activity_parttime	-0.020**
	(0.007)
Activity_self employed	-0.038***
	(0.006)
Activity_unemployed	-0.054***
	(0.007)
Activity_other	0.052***
	(0.003)
Pol. int.	-0.053***
	(0.002)
Sub. hhincome	-0.143***
	(0.002)
Left-right	0.097***
	(0.002)
Party close_no close party	-0.117***
	(0.003)
Party close_nk	-0.061***
	(0.012)
Rel. freedom between	0.008
	(0.023)
Gini Index between	-0.036*
	(0.016)
Gdp between	0.107***

	(0.026)
Pol. effectivity between	0.214***
	(0.028)
Rel. freedom within	0.020
	(0.036)
Gini Index within	-0.098
	(0.064)
Gdp within	0.244***
	(0.061)
Pol. effectivity within	0.126+
	(0.071)
Rel. group_Medium:Rel. freedom between	-0.043***
	(0.011)
Rel. group_Religious:Rel. freedom between	-0.065***
	(0.015)
Rel. group_Medium:Rel. freedom within	-0.034+
	(0.018)
Rel. group_Religious:Rel. freedom within	-0.057*
	(0.027)
SD (Intercept group)	0.270
SD (Rel. group_Medium group)	0.124
SD (Rel. group_Religious group)	0.191
Cor (Intercept~Rel. group_Medium group)	-0.510
Cor (Intercept~Rel. group_Religious group)	-0.513
Cor (Rel. group_Medium~Rel. group_Religious group)	0.879
SD (Observations)	0.871
Num.Obs.	362520
ICC	0.1

- p < 0.1, * p < 0.05, ** p < 0.01, *** p < 0.001

Table 1c: significance test of impact of religious freeedom on average democratic satisfaction

Group	Marginal effect	SE	t
Non-religious	0.0111	0.0199	0.555
Medium religious	-0.0297	0.0173	-1.714
Religious	-0.0523	0.0178	-2.945
Non-Religious, between	0.0083	0.0232	0.359
Medium Religious, between	-0.0349	0.0203	-1.719
Religious, between	-0.0571	0.0209	-2.732
Non-Religious, within	0.0203	0.0364	0.556
Medium Religious, within	-0.0136	0.0312	-0.435
Religious, within	-0.0372	0.0325	-1.144

Table 2a: Impact of Mipex on gap between migrants - natives, linear

	M1	M2	M3	M4	All pol. controls	L2 lagged 1Y
(Intercept)	-0.010	-0.008	0.003	0.004	0.002	0.002
	(0.036)	(0.035)	(0.027)	(0.025)	(0.024)	(0.026)
Mig. back._Migrants	0.129***	0.132***	0.129***	0.128***	0.128***	0.130***
	(0.019)	(0.017)	(0.017)	(0.017)	(0.017)	(0.017)
Gender_male	0.025***	0.025***	0.025***	0.025***	0.025***	0.024***
	(0.004)	(0.004)	(0.004)	(0.004)	(0.004)	(0.004)
Age	-0.049***	-0.049***	-0.049***	-0.049***	-0.049***	-0.049***
	(0.002)	(0.002)	(0.002)	(0.002)	(0.002)	(0.002)
Education	0.022***	0.022***	0.022***	0.022***	0.022***	0.022***
	(0.002)	(0.002)	(0.002)	(0.002)	(0.002)	(0.002)
Activity_parttime	-0.004	-0.004	-0.005	-0.005	-0.005	-0.006
	(0.010)	(0.010)	(0.010)	(0.010)	(0.010)	(0.010)
Activity_self employed	-0.032***	-0.032***	-0.032***	-0.032***	-0.032***	-0.032***
	(0.008)	(0.008)	(0.008)	(0.008)	(0.008)	(0.008)
Activity_unemployed	-0.058***	-0.058***	-0.058***	-0.058***	-0.058***	-0.059***
	(0.009)	(0.009)	(0.009)	(0.009)	(0.009)	(0.009)
Activity_other	0.066***	0.065***	0.065***	0.065***	0.065***	0.066***
	(0.005)	(0.005)	(0.005)	(0.005)	(0.005)	(0.005)
Pol. int.	-0.059***	-0.059***	-0.059***	-0.059***	-0.059***	-0.058***
	(0.002)	(0.002)	(0.002)	(0.002)	(0.002)	(0.002)
Sub. hhincome	-0.148***	-0.148***	-0.147***	-0.147***	-0.147***	-0.148***
	(0.002)	(0.002)	(0.002)	(0.002)	(0.002)	(0.002)
Left-right	0.105***	0.105***	0.105***	0.105***	0.105***	0.105***
	(0.002)	(0.002)	(0.002)	(0.002)	(0.002)	(0.002)
Party close_no close party	-0.126***	-0.126***	-0.126***	-0.126***	-0.126***	-0.127***
	(0.004)	(0.004)	(0.004)	(0.004)	(0.004)	(0.004)
Party close_nk	-0.055**	-0.055**	-0.055**	-0.055**	-0.055**	-0.059***
	(0.017)	(0.017)	(0.017)	(0.017)	(0.017)	(0.018)
Mipex		0.079*	-0.036	-0.070**	-0.078**	-0.073**
		(0.034)	(0.027)	(0.026)	(0.026)	(0.027)
Mig. back._Migrants:Mipex		0.086***	0.087***	0.088***	0.088***	0.085***
		(0.017)	(0.017)	(0.017)	(0.017)	(0.017)
NMR			0.077**	0.079**	0.085**	0.083**
			(0.029)	(0.027)	(0.027)	(0.027)
Gini Index			-0.006	0.011	0.035	0.015
			(0.023)	(0.022)	(0.028)	(0.023)
Gdp			0.260***	0.138***	0.105**	0.148***
			(0.029)	(0.037)	(0.038)	(0.038)

	M1	M2	M3	M4	All pol. controls	L2 lagged 1Y
Pol. effectivity				0.169***	0.090	0.161***
				(0.037)	(0.063)	(0.038)
Pol. stability					0.049	
					(0.032)	
Corruption control					0.095	
					(0.071)	
Pol. voice					0.001	
					(0.046)	
SD (Intercept group)	0.378	0.371	0.284	0.259	0.250	0.266
SD (Mig. back._Migrants group)	0.189	0.169	0.167	0.167	0.168	0.167
Cor (Intercept~Mig. back._Migrants group)	-0.052	-0.177	-0.629	-0.618	-0.575	-0.626
SD (Observations)	0.873	0.873	0.873	0.873	0.873	0.874
Num.Obs.	183962	183962	183962	183962	183962	180142
ICC	0.2	0.2	0.1	0.1	0.1	0.1

- p < 0.1, * p < 0.05, ** p < 0.01, *** p < 0.001

Table 2b: Impact of Mipex on gap between migrants - natives, non linear

	M4	All institutional	L2 lagged 1Y	L2 lagged 2Y
(Intercept)	-0.132***	-0.132***	-0.148***	-0.149***
	(0.032)	(0.033)	(0.032)	(0.039)
Mig. back._Migrants	0.213***	0.213***	0.216***	0.218***
	(0.021)	(0.021)	(0.022)	(0.025)
Gender_male	0.025***	0.025***	0.024***	0.017***
	(0.004)	(0.004)	(0.004)	(0.005)
Age	-0.049***	-0.049***	-0.049***	-0.047***
	(0.002)	(0.002)	(0.002)	(0.002)
Education	0.022***	0.022***	0.023***	0.021***
	(0.002)	(0.002)	(0.002)	(0.003)
Activity_parttime	-0.005	-0.005	-0.006	-0.011
	(0.010)	(0.010)	(0.010)	(0.011)
Activity_self employed	-0.032***	-0.031***	-0.032***	-0.038***
	(0.008)	(0.008)	(0.008)	(0.009)
Activity_unemployed	-0.058***	-0.058***	-0.059***	-0.057***
	(0.009)	(0.009)	(0.009)	(0.010)
Activity_other	0.065***	0.065***	0.066***	0.067***
	(0.005)	(0.005)	(0.005)	(0.005)
Pol. int.	-0.059***	-0.059***	-0.058***	-0.059***
	(0.002)	(0.002)	(0.002)	(0.003)
Sub. hhincome	-0.147***	-0.147***	-0.148***	-0.151***
	(0.002)	(0.002)	(0.002)	(0.003)
Left-right	0.105***	0.105***	0.105***	0.111***
	(0.002)	(0.002)	(0.002)	(0.002)
Party close_no close party	-0.126***	-0.126***	-0.127***	-0.123***
	(0.004)	(0.004)	(0.004)	(0.005)
Party close_nk	-0.055**	-0.055**	-0.060***	-0.056**
	(0.017)	(0.017)	(0.018)	(0.019)
Mipex	-0.148***	-0.160***	-0.153***	-0.148***
	(0.027)	(0.029)	(0.027)	(0.032)
Mipex squared	0.128***	0.128***	0.139***	0.135***
	(0.022)	(0.024)	(0.022)	(0.026)
NMR	0.086***	0.088***	0.085***	0.064*
	(0.026)	(0.026)	(0.025)	(0.029)
Gini Index	0.001	0.006	0.004	0.006
	(0.021)	(0.027)	(0.021)	(0.025)
Gdp	0.161***	0.138***	0.176***	0.184***
	(0.036)	(0.039)	(0.037)	(0.042)
Pol. effectivity	0.152***	0.054	0.143***	0.157***

	M4	All institutional	L2 lagged 1Y	L2 lagged 2Y
	(0.035)	(0.061)	(0.036)	(0.043)
Mig. back._Migrants:Mipex	0.135***	0.135***	0.128***	0.136***
	(0.017)	(0.017)	(0.017)	(0.019)
Mig. back._Migrants:Mipex squared	-0.082***	-0.082***	-0.081***	-0.082***
	(0.015)	(0.015)	(0.015)	(0.017)
Pol. stability		0.003		
		(0.034)		
Corruption control		0.102		
		(0.068)		
Pol. voice		0.027		
		(0.045)		
SD (Intercept group)	0.228	0.224	0.228	0.247
SD (Mig. back._Migrants group)	0.145	0.145	0.145	0.154
Cor (Intercept~Mig. back._Migrants group)	-0.501	-0.482	-0.507	-0.574
SD (Observations)	0.873	0.873	0.874	0.877
Num.Obs.	183962	183962	180142	151319
ICC	0.1	0.1	0.1	0.1

- p < 0.1, * p < 0.05, ** p < 0.01, *** p < 0.001

Table 2c: Impact of Mipex on gap between migrants - natives, non linear, within-between

(Intercept)	-0.174***
	(0.036)
Mig. back._Migrants	0.218***
	(0.024)
Gender_male	0.025***
	(0.004)
Age	-0.049***
	(0.002)
Education	0.022***
	(0.002)
Activity_parttime	-0.005
	(0.010)
Activity_self employed	-0.032***
	(0.008)
Activity_unemployed	-0.058***
	(0.009)
Activity_other	0.065***
	(0.005)
Pol. int.	-0.059***
	(0.002)
Sub. hhincome	-0.147***
	(0.002)
Left-right	0.105***
	(0.002)
Party close_no close party	-0.126***
	(0.004)
Party close_nk	-0.055**
	(0.017)
Mipex between	-0.161***
	(0.028)
Mipex between squared	0.148***
	(0.024)
Gini Index between	0.004
	(0.021)
Gdp between	0.163***
	(0.040)
Pol. effectivity between	0.142***
	(0.041)

NMR between	0.097***
	(0.029)
Mipex within	-0.084
	(0.127)
Mipex within squared	0.860*
	(0.385)
Gini Index within	0.022
	(0.130)
Gdp within	0.183+
	(0.099)
Pol. effectivity within	0.118
	(0.107)
NMR within	0.080
	(0.055)
Mig. back._Migrants:Mipex between	0.140***
	(0.018)
Mig. back._Migrants:Mipex between squared	-0.086***
	(0.015)
Mig. back._Migrants:Mipex within	0.036
	(0.089)
Mig. back._Migrants:Mipex within squared	-0.124
	(0.265)
SD (Intercept group)	0.226
SD (Mig. back._Migrants group)	0.146
Cor (Intercept~Mig. back._Migrants group)	-0.504
SD (Observations)	0.873
Num.Obs.	183962
ICC	0.1

- p < 0.1, * p < 0.05, ** p < 0.01, *** p < 0.001

Table 2d: significance test of impact of Mipex on average democratic satisfaction

Group	Marginal effect	SE	T
Natives, overall	-0.12621....	0.0192	-6.55903....
Migrants, overall	-0.07283....	0.0219	-3.32790....
Natives, between	-0.13503....	0.0192	-7.02014....
Migrants, between	-0.08034....	0.0222	-3.61439....
Natives, within	-0.07657....	0.1056	-0.72493....
Migrants, within	-0.16466....	0.2876	-0.57262....

Table 3a: Impact of Mipex on xenophobes-non xenophobes gap

	M1	M2	M3	M4	All pol. controls	L2 lagged 1Y
(Intercept)	-0.010	-0.008	0.003	0.004	0.002	0.002
	(0.036)	(0.035)	(0.027)	(0.025)	(0.024)	(0.026)
Mig. back._Migrants	0.129***	0.132***	0.129***	0.128***	0.128***	0.130***
	(0.019)	(0.017)	(0.017)	(0.017)	(0.017)	(0.017)
Gender_male	0.025***	0.025***	0.025***	0.025***	0.025***	0.024***
	(0.004)	(0.004)	(0.004)	(0.004)	(0.004)	(0.004)
Age	-0.049***	-0.049***	-0.049***	-0.049***	-0.049***	-0.049***
	(0.002)	(0.002)	(0.002)	(0.002)	(0.002)	(0.002)
Education	0.022***	0.022***	0.022***	0.022***	0.022***	0.022***
	(0.002)	(0.002)	(0.002)	(0.002)	(0.002)	(0.002)
Activity_parttime	-0.004	-0.004	-0.005	-0.005	-0.005	-0.006
	(0.010)	(0.010)	(0.010)	(0.010)	(0.010)	(0.010)
Activity_self employed	-0.032***	-0.032***	-0.032***	-0.032***	-0.032***	-0.032***
	(0.008)	(0.008)	(0.008)	(0.008)	(0.008)	(0.008)
Activity_unemployed	-0.058***	-0.058***	-0.058***	-0.058***	-0.058***	-0.059***
	(0.009)	(0.009)	(0.009)	(0.009)	(0.009)	(0.009)
Activity_other	0.066***	0.065***	0.065***	0.065***	0.065***	0.066***
	(0.005)	(0.005)	(0.005)	(0.005)	(0.005)	(0.005)
Pol. int.	-0.059***	-0.059***	-0.059***	-0.059***	-0.059***	-0.058***
	(0.002)	(0.002)	(0.002)	(0.002)	(0.002)	(0.002)
Sub. hhincome	-0.148***	-0.148***	-0.147***	-0.147***	-0.147***	-0.148***
	(0.002)	(0.002)	(0.002)	(0.002)	(0.002)	(0.002)
Left-right	0.105***	0.105***	0.105***	0.105***	0.105***	0.105***
	(0.002)	(0.002)	(0.002)	(0.002)	(0.002)	(0.002)
Party close_no close party	-0.126***	-0.126***	-0.126***	-0.126***	-0.126***	-0.127***
	(0.004)	(0.004)	(0.004)	(0.004)	(0.004)	(0.004)
Party close_nk	-0.055**	-0.055**	-0.055**	-0.055**	-0.055**	-0.059***
	(0.017)	(0.017)	(0.017)	(0.017)	(0.017)	(0.018)
Mipex		0.079*	-0.036	-0.070**	-0.078**	-0.073**
		(0.034)	(0.027)	(0.026)	(0.026)	(0.027)
Mig. back._Migrants:Mipex		0.086***	0.087***	0.088***	0.088***	0.085***
		(0.017)	(0.017)	(0.017)	(0.017)	(0.017)
NMR			0.077**	0.079**	0.085**	0.083**
			(0.029)	(0.027)	(0.027)	(0.027)
Gini Index			-0.006	0.011	0.035	0.015
			(0.023)	(0.022)	(0.028)	(0.023)

	M1	M2	M3	M4	All pol. controls	L2 lagged 1Y
Gdp			0.260***	0.138***	0.105**	0.148***
			(0.029)	(0.037)	(0.038)	(0.038)
Pol. effectivity				0.169***	0.090	0.161***
				(0.037)	(0.063)	(0.038)
Pol. stability					0.049	
					(0.032)	
Corruption control					0.095	
					(0.071)	
Pol. voice					0.001	
					(0.046)	
SD (Intercept group)	0.378	0.371	0.284	0.259	0.250	0.266
SD (Mig. back._Migrants group)	0.189	0.169	0.167	0.167	0.168	0.167
Cor (Intercept~Mig. back._Migrants group)	-0.052	-0.177	-0.629	-0.618	-0.575	-0.626
SD (Observations)	0.873	0.873	0.873	0.873	0.873	0.874
Num.Obs.	183962	183962	183962	183962	183962	180142
ICC	0.2	0.2	0.1	0.1	0.1	0.1

- p < 0.1, * p < 0.05, ** p < 0.01, *** p < 0.001

Table 3b: Impact of Mipex on xenophobes-non xenophobes gap, separating country-time effects

(Intercept)	0.235***
	(0.021)
Xenophobia_Medium	-0.200***
	(0.011)
Xenophobia_Xenophob	-0.634***
	(0.023)
Mig. back._Migrants	0.102***
	(0.005)
Gender_male	0.031***
	(0.004)
Age	-0.037***
	(0.002)
Education	-0.008***
	(0.002)
Activity_parttime	-0.008
	(0.009)
Activity_self employed	-0.043***
	(0.008)
Activity_unemployed	-0.049***
	(0.009)
Activity_other	0.054***
	(0.004)
Pol. int.	-0.035***
	(0.002)
Sub. hhincome	-0.123***
	(0.002)
Left-right	0.125***
	(0.002)
Party close_no close party	-0.111***
	(0.004)
Party close_nk	-0.045**
	(0.016)
Mipex between	-0.040+
	(0.023)
Gini Index between	-0.042*
	(0.020)
Gdp between	0.109**

	(0.034)
Pol. effectivity between	0.212***
	(0.037)
Mipex within	0.008
	(0.117)
Gini Index within	-0.008
	(0.117)
Gdp within	0.189**
	(0.070)
Pol. effectivity within	0.167+
	(0.087)
Xenophobia_Medium:Mipex between	-0.029*
	(0.011)
Xenophobia_Xenophob:Mipex between	-0.105***
	(0.023)
Xenophobia_Medium:Mipex within	-0.101
	(0.062)
Xenophobia_Xenophob:Mipex within	-0.074
	(0.129)
SD (Intercept group)	0.244
SD (Xenophobia_Medium group)	0.120
SD (Xenophobia_Xenophob group)	0.266
Cor (Intercept~Xenophobia_Medium group)	-0.434
Cor (Intercept~Xenophobia_Xenophob group)	-0.320
Cor (Xenophobia_Medium~Xenophobia_Xenophob group)	0.861
SD (Observations)	0.851
Num.Obs.	212375
ICC	0.1

- $p < 0.1$, * $p < 0.05$, ** $p < 0.01$, *** $p < 0.001$

Table 3c: significance test of impact of Mipex on average democratic satisfaction

Group	Marginal effect	SE	t
Non-xenophobes	-0.0341	0.0225	-1.516
Medium xenophobes	-0.0653	0.0204	-3.2085
Xenophobes	-0.1381	0.0264	-5.2397
Non-xenophobes, between	-0.0399	0.0233	-1.7086
Medium xenophobes, between	-0.0686	0.0213	-3.2214
xenophobes, between	-0.1445	0.0274	-5.2662
Non-xenophobes, within	0.0081	0.1169	0.0694
Medium xenophobes, within	-0.0931	0.1047	-0.8894
Xenophobes, within	-0.0658	0.1426	-0.4613

Table 4a: Impact of civ. lib. index on homophobes - non homophobes gap

	M1	M2	M3	M4	L2 lagged 1Y	L2 lagged 2Y
(Intercept)	-0.030	-0.019	-0.010	-0.005	-0.005	-0.005
	(0.028)	(0.023)	(0.019)	(0.017)	(0.017)	(0.017)
Homophobia_medium	0.021*	0.020*	0.020*	0.020*	0.021*	0.020*
	(0.009)	(0.008)	(0.008)	(0.008)	(0.008)	(0.008)
Homophobia_Homophobic	-0.073***	-0.075***	-0.075***	-0.074***	-0.073***	-0.074***
	(0.016)	(0.015)	(0.015)	(0.015)	(0.015)	(0.015)
Gender_male	0.030***	0.030***	0.030***	0.030***	0.030***	0.030***
	(0.003)	(0.003)	(0.003)	(0.003)	(0.003)	(0.003)
Mig. back._Migrants	0.135***	0.135***	0.134***	0.134***	0.134***	0.134***
	(0.004)	(0.004)	(0.004)	(0.004)	(0.004)	(0.004)
Age	-0.044***	-0.044***	-0.044***	-0.044***	-0.044***	-0.044***
	(0.002)	(0.002)	(0.002)	(0.002)	(0.002)	(0.002)
Education	0.015***	0.015***	0.014***	0.014***	0.014***	0.014***
	(0.002)	(0.002)	(0.002)	(0.002)	(0.002)	(0.002)
Activity_parttime	-0.011	-0.011	-0.011	-0.011	-0.011	-0.011
	(0.007)	(0.007)	(0.007)	(0.007)	(0.007)	(0.007)
Activity_self employed	-0.041***	-0.041***	-0.040***	-0.040***	-0.040***	-0.040***
	(0.006)	(0.006)	(0.006)	(0.006)	(0.006)	(0.006)
Activity_unemployed	-0.056***	-0.056***	-0.056***	-0.056***	-0.056***	-0.056***
	(0.007)	(0.007)	(0.007)	(0.007)	(0.007)	(0.007)
Activity_other	0.057***	0.057***	0.056***	0.056***	0.056***	0.056***
	(0.003)	(0.003)	(0.003)	(0.003)	(0.003)	(0.003)
Pol. int.	-0.054***	-0.054***	-0.053***	-0.053***	-0.053***	-0.053***
	(0.002)	(0.002)	(0.002)	(0.002)	(0.002)	(0.002)
Sub. hhincome	-0.141***	-0.141***	-0.140***	-0.140***	-0.140***	-0.140***

	M1	M2	M3	M4	L2 lagged 1Y	L2 lagged 2Y
	(0.002)	(0.002)	(0.002)	(0.002)	(0.002)	(0.002)
Left-right	0.105***	0.105***	0.105***	0.105***	0.105***	0.105***
	(0.002)	(0.002)	(0.002)	(0.002)	(0.002)	(0.002)
Party close_no close party	-0.120***	-0.120***	-0.120***	-0.120***	-0.120***	-0.120***
	(0.003)	(0.003)	(0.003)	(0.003)	(0.003)	(0.003)
Party close_nk	-0.068***	-0.068***	-0.068***	-0.068***	-0.068***	-0.068***
	(0.012)	(0.012)	(0.012)	(0.012)	(0.012)	(0.012)
Civ. liberty		0.238***	0.097***	0.029	0.009	0.023
		(0.022)	(0.022)	(0.022)	(0.023)	(0.023)
Homophobia_medium:Civ. liberty		-0.065***	-0.065***	-0.066***	-0.063***	-0.064***
		(0.008)	(0.008)	(0.008)	(0.008)	(0.008)
Homophobia_Homophobic:Civ. liberty		-0.080***	-0.081***	-0.082***	-0.077***	-0.077***
		(0.014)	(0.014)	(0.014)	(0.014)	(0.014)
Gdp			0.233***	0.136***	0.129***	0.130***
			(0.018)	(0.022)	(0.022)	(0.024)
Gini Index			-0.035*	-0.033*	-0.036*	-0.037*
			(0.018)	(0.016)	(0.016)	(0.017)
Pol. effectivity				0.175***	0.190***	0.174***
				(0.026)	(0.026)	(0.028)
SD (Intercept group)	0.427	0.352	0.279	0.254	0.255	0.261
SD (Homophobia_medium group)	0.124	0.107	0.107	0.107	0.108	0.108
SD (Homophobia_Homophobic group)	0.223	0.210	0.211	0.211	0.213	0.212
Cor (Intercept~Homophobia_medium group)	-0.594	-0.428	-0.415	-0.398	-0.416	-0.412
Cor (Intercept~Homophobia_Homophobic group)	-0.476	-0.356	-0.458	-0.442	-0.451	-0.447
Cor (Homophobia_medium~Homophobia_Homophobic group)	0.849	0.835	0.835	0.836	0.841	0.840
SD (Observations)	0.872	0.872	0.872	0.872	0.872	0.872
Num.Obs.	360122	360122	360122	360122	360122	360122

	M1	M2	M3	M4	L2 lagged 1Y	L2 lagged 2Y
ICC	0.2	0.1	0.1	0.1	0.1	0.1

- p < 0.1, * p < 0.05, ** p < 0.01, *** p < 0.001

Table 4b: Impact of civ. lib. index on homophobes - non homophobes gap, separating country-time effects

(Intercept)	-0.005
	(0.017)
Homophobia_medium	0.021**
	(0.008)
Homophobia_Homophobic	-0.071***
	(0.015)
Gender_male	0.030***
	(0.003)
Mig. back._Migrants	0.134***
	(0.004)
Age	-0.044***
	(0.002)
Education	0.014***
	(0.002)
Activity_parttime	-0.011
	(0.007)
Activity_self employed	-0.040***
	(0.006)
Activity_unemployed	-0.056***
	(0.007)
Activity_other	0.056***
	(0.003)
Pol. int.	-0.053***
	(0.002)
Sub. hhincome	-0.140***
	(0.002)
Left-right	0.105***
	(0.002)
Party close_no close party	-0.120***
	(0.003)
Party close_nk	-0.068***
	(0.012)
Civ. liberty between	0.043+
	(0.025)
Gini Index between	-0.023
	(0.017)
Gdp between	0.111***

	(0.025)
Pol. effectivity between	0.188***
	(0.030)
Civ. liberty within	0.010
	(0.050)
Gini Index within	-0.103
	(0.063)
Gdp within	0.209***
	(0.060)
Pol. effectivity within	0.142*
	(0.069)
Homophobia_medium:Civ. liberty between	-0.066***
	(0.009)
Homophobia_Homophobic:Civ. liberty between	-0.072***
	(0.015)
Homophobia_medium:Civ. liberty within	-0.063**
	(0.024)
Homophobia_Homophobic:Civ. liberty within	-0.175***
	(0.042)
SD (Intercept group)	0.252
SD (Homophobia_medium group)	0.107
SD (Homophobia_Homophobic group)	0.208
Cor (Intercept~Homophobia_medium group)	-0.392
Cor (Intercept~Homophobia_Homophobic group)	-0.450
Cor (Homophobia_medium~Homophobia_Homophobic group)	0.851
SD (Observations)	0.872
Num.Obs.	360122
ICC	0.1

- p < 0.1, * p < 0.05, ** p < 0.01, *** p < 0.001

Table 4c: significance test of impact of civ. lib. index on average democratic satisfaction

Group	Marginal effect	SE	t
Non-homophobes	0.0286	0.0223	1.281
Medium homophobes	-0.03702	0.0211	-1.754
Homophobes	-0.05335	0.0219	-2.438
Non-homophobes, within	0.00963	0.0498	0.193
Medium homophobes, within	-0.0536	0.0454	-1.181
Homophobes, within	-0.1651	0.0485	-3.404
Non-homophobes, within	0.00963	0.0498	0.193
Medium homophobes, within	-0.0536	0.0454	-1.181
Homophobes, within	-0.1651	0.0485	-3.404

Table 5a: Impact of gender equality index on gender gap

	M1	M2	M3	M4	L2 lagged 1Y	L2 lagged 2Y
(Intercept)	0.108***	0.118***	0.122***	0.127***	0.127***	0.127***
	(0.025)	(0.022)	(0.018)	(0.017)	(0.017)	(0.017)
Gender_male	0.020***	0.020***	0.020***	0.020***	0.020***	0.020***
	(0.005)	(0.005)	(0.005)	(0.005)	(0.005)	(0.005)
Age	-0.046***	-0.046***	-0.046***	-0.046***	-0.046***	-0.046***
	(0.002)	(0.002)	(0.002)	(0.002)	(0.002)	(0.002)
Education	0.014***	0.014***	0.014***	0.014***	0.014***	0.014***
	(0.002)	(0.002)	(0.002)	(0.002)	(0.002)	(0.002)
Activity_parttime	-0.011	-0.011	-0.012+	-0.012+	-0.012+	-0.012+
	(0.007)	(0.007)	(0.007)	(0.007)	(0.007)	(0.007)
Activity_self employed	-0.041***	-0.041***	-0.041***	-0.041***	-0.041***	-0.041***
	(0.006)	(0.006)	(0.006)	(0.006)	(0.006)	(0.006)
Activity_unemployed	-0.055***	-0.055***	-0.055***	-0.055***	-0.055***	-0.055***
	(0.007)	(0.007)	(0.007)	(0.007)	(0.007)	(0.007)
Activity_other	0.055***	0.055***	0.055***	0.055***	0.055***	0.055***
	(0.003)	(0.003)	(0.003)	(0.003)	(0.003)	(0.003)
Mig. back._Migrants	0.128***	0.128***	0.128***	0.128***	0.128***	0.128***
	(0.004)	(0.004)	(0.004)	(0.004)	(0.004)	(0.004)
Pol. int.	-0.054***	-0.054***	-0.054***	-0.054***	-0.054***	-0.054***
	(0.002)	(0.002)	(0.002)	(0.002)	(0.002)	(0.002)
Sub. hhincome	-0.143***	-0.143***	-0.143***	-0.143***	-0.143***	-0.143***
	(0.002)	(0.002)	(0.002)	(0.002)	(0.002)	(0.002)
Left-right	0.106***	0.106***	0.106***	0.106***	0.106***	0.106***
	(0.001)	(0.001)	(0.001)	(0.001)	(0.001)	(0.001)
clsprty	-0.119***	-0.119***	-0.119***	-0.119***	-0.119***	-0.119***
	(0.003)	(0.003)	(0.003)	(0.003)	(0.003)	(0.003)
Gender Equality		0.187***	0.052*	-0.007	-0.014	-0.010
		(0.021)	(0.020)	(0.021)	(0.021)	(0.022)
Gender_male:Gender Equality		0.008	0.008	0.008	0.009+	0.009+
		(0.005)	(0.005)	(0.005)	(0.005)	(0.005)
Gini Index			-0.040*	-0.030+	-0.031+	-0.035*
			(0.018)	(0.017)	(0.017)	(0.018)
Gdp			0.236***	0.128***	0.122***	0.124***
			(0.018)	(0.023)	(0.023)	(0.025)
Pol. effectivity				0.192***	0.200***	0.186***
				(0.025)	(0.026)	(0.027)
SD (Intercept group)	0.382	0.333	0.260	0.249	0.250	0.253

	M1	M2	M3	M4	L2 lagged 1Y	L2 lagged 2Y
SD (Gender_male group)	0.064	0.064	0.064	0.064	0.064	0.064
Cor (Intercept~Gender_male group)	0.131	0.082	-0.131	-0.364	-0.361	-0.326
SD (Observations)	0.874	0.874	0.874	0.874	0.874	0.874
Num.Obs.	363186	363186	363186	363186	363186	363186
ICC	0.2	0.1	0.1	0.1	0.1	0.1

- p < 0.1, * p < 0.05, ** p < 0.01, *** p < 0.001

Table 5b: Impact of gender equality index on gender gap separating country-time effects

(Intercept)	0.144***
	(0.017)
Gender_male	0.020***
	(0.005)
Age	-0.048***
	(0.002)
Education	0.016***
	(0.002)
Activity_parttime	-0.012+
	(0.007)
Activity_self employed	-0.041***
	(0.006)
Activity_unemployed	-0.051***
	(0.007)
Activity_other	0.054***
	(0.003)
Pol. int.	-0.054***
	(0.002)
Sub. Hhincome	-0.139***
	(0.002)
Left-right	0.105***
	(0.001)
Clsprty	-0.117***
	(0.003)
Gender Equality between	-0.015
	(0.023)
Gini Index between	-0.018
	(0.017)
Gdp between	0.108***
	(0.026)
Pol. effectivity between	0.220***
	(0.030)
Gender Equality within	0.003
	(0.054)
Gini Index within	-0.109+
	(0.065)
Gdp within	0.243***
	(0.065)

Pol. effectivity within	0.149*
	(0.072)
Gender_male:Gender Equality between	0.014**
	(0.005)
Gender_male:Gender Equality within	-0.047**
	(0.016)
SD (Intercept group)	0.246
SD (Gender_male group)	0.062
Cor (Intercept~Gender_male group)	-0.356
SD (Observations)	0.875
Num.Obs.	363186
ICC	0.1

- p < 0.1, * p < 0.05, ** p < 0.01, *** p < 0.001

Table 5c: significance test of impact of gender equality index on average democratic satisfaction

Group	Marginal effect	SE	t
female	-0.007263	0.0206	-0.3531
male	0.00048	0.0198	0.0242
female, between	-0.014762	0.0229	-0.6447
male, between	-0.000914	0.0222	-0.0412
female, within	0.003491	0.0538	0.0648
male, within	-0.04304	0.0512	-0.8406

Table 6a:distribution of continuous variables, subsample religiosity

	Mean	SD	Min	Max
Rel. attitude	4.629	3.031	0.000	10.000
Rel. attendence	5.445	1.518	1.000	7.000
Pray	4.708	2.412	1.000	7.000
Rel. freedom	0.868	0.122	0.380	1.000
dem. satisfaction	5.318	2.496	0.000	10.000
age	48.533	18.155	14.000	95.000
education	12.684	3.985	0.000	30.000
pol. interest	2.524	0.887	1.000	4.000
sub. hhincome	1.992	0.860	1.000	4.000
left-right	5.138	2.237	0.000	10.000
gdp	41638.988	14394.714	11227.966	107634.837
gini index	31.441	4.028	24.400	41.900
effectivity	1.251	0.655	-0.867	2.287
total n = 365039				

Table 6b:distribution of categorical variables, subsample religiosity

		N	%
Rel. group	Non-reliegious	74657	20.452
	Medium	217383	59.551
	Religious	72999	19.998
partycloseness	close party	191603	52.488
	no close party	167595	45.912
	nk	5841	1.600
activity	fulltime	136681	37.443
	parttime	18935	5.187
	self employed	25866	7.086
	unemployed	19075	5.225
	other	164482	45.059
gender	female	189678	51.961
	male	175361	48.039
Mig.back	Natives	306844	84.058
	Migrants	58195	15.942
total n = 365039			

Table 6c: Surveys used in subsample religiosity

Ccode	Year	n
ALB	2012	1029
AUT	2003	1722
AUT	2005	1752
AUT	2007	1760
AUT	2015	1569
AUT	2016	1711
AUT	2018	2080
BEL	2002	1456
BEL	2004	1538
BEL	2006	1697
BEL	2008	1648
BEL	2011	1591
BEL	2012	1782
BEL	2014	1675
BEL	2016	1680
BEL	2018	1658
BGR	2006	871
BGR	2009	1497
BGR	2011	1705
BGR	2013	1668
BGR	2018	1201
BGR	2021	2242
CHE	2002	1775
CHE	2004	1861
CHE	2006	1631
CHE	2008	1600
CHE	2010	1368
CHE	2012	1356
CHE	2014	1371
CHE	2016	1365
CHE	2018	1333
CHE	2021	1360
CYP	2006	785
CYP	2008	967
CYP	2011	710
CYP	2012	780
CYP	2018	547
CZE	2002	1037
CZE	2004	1982

Ccode	Year	n
CZE	2009	1665
CZE	2011	2001
CZE	2013	1474
CZE	2015	1732
CZE	2016	1928
CZE	2019	1942
CZE	2021	1978
DEU	2003	2619
DEU	2004	2416
DEU	2006	2444
DEU	2008	2446
DEU	2010	2725
DEU	2012	2731
DEU	2014	2844
DEU	2016	2698
DEU	2018	2190
DNK	2002	1323
DNK	2004	1310
DNK	2006	1364
DNK	2008	1506
DNK	2010	1446
DNK	2013	1528
DNK	2014	1398
DNK	2018	1436
ESP	2002	1187
ESP	2004	1324
ESP	2006	1490
ESP	2008	1944
ESP	2011	1623
ESP	2013	1652
ESP	2015	1554
ESP	2017	1601
ESP	2019	1343
EST	2004	1373
EST	2007	1014
EST	2009	1206
EST	2010	1438
EST	2012	1833
EST	2014	1606
EST	2016	1684

Ccode	Year	n
EST	2018	1665
EST	2021	1389
FIN	2002	1813
FIN	2004	1855
FIN	2006	1767
FIN	2008	2026
FIN	2010	1730
FIN	2012	2029
FIN	2014	1922
FIN	2016	1823
FIN	2018	1626
FIN	2021	1490
FRA	2006	1823
FRA	2008	1885
FRA	2010	1608
FRA	2013	1807
FRA	2014	1747
FRA	2016	1858
FRA	2018	1728
FRA	2021	1667
GBR	2002	1772
GBR	2004	1624
GBR	2006	1996
GBR	2008	2015
GBR	2010	1899
GBR	2012	1792
GBR	2014	1928
GBR	2016	1682
GBR	2018	1906
GRC	2003	1907
GRC	2005	1889
GRC	2009	1643
GRC	2011	1905
HRV	2009	983
HRV	2011	1153
HRV	2019	1537
HRV	2021	1306
HUN	2002	1314
HUN	2005	1158
HUN	2006	1163

Ccode	Year	n
HUN	2009	1155
HUN	2010	1278
HUN	2012	1545
HUN	2015	1314
HUN	2017	1220
HUN	2019	1272
HUN	2021	1467
IRL	2003	1532
IRL	2005	1748
IRL	2007	1275
IRL	2009	1580
IRL	2011	2116
IRL	2013	2169
IRL	2014	1797
IRL	2017	2132
IRL	2019	1814
ISL	2005	497
ISL	2012	658
ISL	2017	790
ISL	2019	769
ISL	2021	803
ISR	2002	2051
ISR	2008	2012
ISR	2011	1685
ISR	2012	2087
ISR	2015	2189
ISR	2016	2189
ITA	2003	871
ITA	2006	1196
ITA	2013	713
ITA	2017	1552
ITA	2019	1838
LTU	2011	971
LTU	2013	1349
LTU	2015	1379
LTU	2017	1338
LTU	2019	1146
LTU	2021	1093
LUX	2003	1002
LUX	2004	1244

Ccode	Year	n
LVA	2007	1207
LVA	2009	1464
LVA	2019	591
MNE	2019	732
NLD	2002	2178
NLD	2004	1708
NLD	2006	1729
NLD	2008	1646
NLD	2010	1683
NLD	2012	1731
NLD	2014	1740
NLD	2016	1534
NLD	2018	1490
NLD	2021	1360
NOR	2002	1941
NOR	2004	1704
NOR	2006	1668
NOR	2008	1482
NOR	2010	1456
NOR	2012	1584
NOR	2014	1375
NOR	2016	1477
NOR	2019	1306
NOR	2021	1341
POL	2002	1608
POL	2004	1287
POL	2006	1320
POL	2008	1235
POL	2010	1373
POL	2012	1473
POL	2015	1182
POL	2016	1264
POL	2018	1055
PRT	2002	1092
PRT	2005	1277
PRT	2007	1382
PRT	2009	1416
PRT	2011	1288
PRT	2013	1376
PRT	2015	1045

Ccode	Year	n
PRT	2017	1119
PRT	2019	896
PRT	2021	1364
ROU	2006	1312
ROU	2009	1188
RUS	2006	1104
RUS	2008	1310
RUS	2011	1426
RUS	2012	1545
RUS	2017	1312
SRB	2018	1183
SVK	2004	1111
SVK	2006	1416
SVK	2008	1471
SVK	2010	1469
SVK	2012	1556
SVK	2019	906
SVK	2021	1083
SVN	2002	1102
SVN	2004	950
SVN	2006	1016
SVN	2008	952
SVN	2010	872
SVN	2012	843
SVN	2014	892
SVN	2016	1027
SVN	2018	1024
SVN	2020	1036
SWE	2002	1798
SWE	2004	1777
SWE	2006	1746
SWE	2008	1712
SWE	2010	1405
SWE	2012	1708
SWE	2014	1656
SWE	2016	1430
SWE	2018	1435
TUR	2006	1409
TUR	2009	1780
UKR	2005	1076

Ccode	Year	n
UKR	2006	1023
UKR	2009	842
UKR	2011	866
UKR	2013	1049

total n = 365039

Table 7a:distribution of continuous variables, subsample migration

	Mean	SD	Min	Max
economic	4.992	2.398	0.000	10.000
cultural	5.598	2.496	0.000	10.000
overall	4.995	2.268	0.000	10.000
Mipex	56.898	14.377	34.000	88.000
dem. satisfaction	5.307	2.470	0.000	10.000
age	48.968	18.190	14.000	95.000
education	12.817	3.983	0.000	30.000
pol. interest	2.523	0.886	1.000	4.000
sub. hhincome	1.988	0.853	1.000	4.000
left-right	5.126	2.203	0.000	10.000
gdp	42470.446	12402.979	18661.480	77749.202
gini index	31.288	3.738	24.400	41.400
effectivity	1.321	0.516	-0.191	2.241
total n = 185198				

Table 7b:distribution of categorical variables, subsample migration

		N	%
partycloseness	close party	96040	51.858
	no close party	86535	46.726
	nk	2623	1.416
activity	fulltime	69795	37.687
	parttime	9823	5.304
	self employed	13276	7.169
	unemployed	10301	5.562
	other	82003	44.279
gender	female	96201	51.945
	male	88997	48.055
Mig.back	Natives	155753	84.101
	Migrants	29445	15.899
total n = 185198			

Table 7c: Surveys used in subsample migration

Ccode	Year	n
AUT	2015	1611
AUT	2016	1785
BEL	2008	1654
BEL	2011	1593
BEL	2012	1784
BEL	2014	1679
BEL	2016	1680
BGR	2011	1765
BGR	2013	1714
CHE	2008	1621
CHE	2010	1383
CHE	2012	1379
CHE	2014	1394
CHE	2016	1389
CYP	2008	981
CYP	2011	757
CYP	2012	791
CZE	2009	1744
CZE	2011	2047
CZE	2013	1599
CZE	2015	1782
CZE	2016	1964
DEU	2008	2482
DEU	2010	2759
DEU	2012	2755
DEU	2014	2866
DEU	2016	2711
DNK	2008	1513
DNK	2010	1455
DNK	2013	1534
DNK	2014	1412
ESP	2008	1968
ESP	2011	1633
ESP	2013	1656
ESP	2015	1575
ESP	2017	1620
EST	2007	1034
EST	2009	1222
EST	2010	1448

Ccode	Year	n
EST	2012	1858
EST	2014	1636
EST	2016	1692
FIN	2008	2033
FIN	2010	1734
FIN	2012	2039
FIN	2014	1932
FIN	2016	1828
FRA	2008	1896
FRA	2010	1613
FRA	2013	1815
FRA	2014	1754
FRA	2016	1865
GBR	2008	2027
GBR	2010	1915
GBR	2012	1809
GBR	2014	1940
GBR	2016	1687
GRC	2009	1677
GRC	2011	1923
HUN	2009	1170
HUN	2010	1294
HUN	2012	1599
HUN	2015	1342
HUN	2017	1252
IRL	2007	1297
IRL	2009	1583
IRL	2011	2128
IRL	2013	2190
IRL	2014	1821
IRL	2017	2148
ISL	2017	793
ISR	2015	2249
ISR	2016	2221
ITA	2013	722
ITA	2017	1615
LTU	2011	996
LTU	2013	1411
LTU	2015	1452
LTU	2017	1433

Ccode	Year	n
LVA	2007	1236
LVA	2009	1553
NLD	2008	1653
NLD	2010	1692
NLD	2012	1736
NLD	2014	1745
NLD	2016	1540
NOR	2008	1492
NOR	2010	1469
NOR	2012	1591
NOR	2014	1385
NOR	2016	1480
POL	2008	1295
POL	2010	1439
POL	2012	1545
POL	2015	1241
POL	2016	1349
PRT	2007	1489
PRT	2009	1501
PRT	2011	1376
PRT	2013	1436
PRT	2015	1055
PRT	2017	1125
RUS	2017	1427
SVK	2008	1486
SVK	2010	1501
SVK	2012	1590
SVN	2008	989
SVN	2010	910
SVN	2012	851
SVN	2014	907
SVN	2016	1039
SWE	2008	1719
SWE	2010	1409
SWE	2012	1712
SWE	2014	1668
SWE	2016	1439

total n = 185198

Table 8a:distribution of continuous variables, subsample xenophobia

	Mean	SD	Min	Max
economic	5.065	2.413	0.000	10.000
cultural	5.590	2.521	0.000	10.000
overall	5.036	2.291	0.000	10.000
Mipex	56.865	14.145	34.000	88.000
dem. satisfaction	5.354	2.470	0.000	10.000
age	49.083	18.137	14.000	95.000
education	12.961	3.967	0.000	30.000
pol. interest	2.514	0.887	1.000	4.000
sub. hhincome	1.955	0.843	1.000	4.000
left-right	5.110	2.201	0.000	10.000
gdp	43470.826	12844.495	17452.803	86650.005
gini index	31.201	3.632	24.400	41.400
effectivity	1.309	0.518	-0.191	2.241
total n = 213512				

Table 8b:distribution of categorical variables, subsample xenophobia

		N	%
xenophobia	Non-xenophob	45029	21.090
	Medium	126047	59.035
	Xenophob	42436	19.875
partycloseness	close party	111289	52.123
	no close party	99408	46.559
	nk	2815	1.318
activity	fulltime	81492	38.167
	parttime	11299	5.292
	self employed	15415	7.220
	unemployed	11401	5.340
	other	93905	43.981
gender	female	110186	51.606
	male	103326	48.394
Mig.back	Natives	179518	84.079
	Migrants	33994	15.921
total n = 213512			

Table 8c: Surveys used in subsample xenophobia

Ccode	Year	n
AUT	2015	1520
AUT	2016	1696
AUT	2018	2046
BEL	2008	1625
BEL	2011	1576
BEL	2012	1770
BEL	2014	1666
BEL	2016	1669
BEL	2018	1637
BGR	2011	1429
BGR	2013	1399
BGR	2018	1086
CHE	2008	1547
CHE	2010	1346
CHE	2012	1332
CHE	2014	1353
CHE	2016	1341
CHE	2018	1278
CYP	2008	958
CYP	2011	727
CYP	2012	773
CYP	2018	523
CZE	2009	1595
CZE	2011	1889
CZE	2013	1414
CZE	2015	1643
CZE	2016	1884
CZE	2019	1909
DEU	2008	2399
DEU	2010	2619
DEU	2012	2686
DEU	2014	2804
DEU	2016	2666
DEU	2018	2177
DNK	2008	1459
DNK	2010	1407
DNK	2013	1481
DNK	2014	1381
DNK	2018	1407

Ccode	Year	n
ESP	2008	1825
ESP	2011	1564
ESP	2013	1607
ESP	2015	1445
ESP	2017	1514
ESP	2019	1251
EST	2007	939
EST	2009	1165
EST	2010	1363
EST	2012	1736
EST	2014	1553
EST	2016	1660
EST	2018	1639
FIN	2008	2018
FIN	2010	1710
FIN	2012	2017
FIN	2014	1903
FIN	2016	1807
FIN	2018	1615
FRA	2008	1858
FRA	2010	1595
FRA	2013	1797
FRA	2014	1722
FRA	2016	1838
FRA	2018	1707
GBR	2008	1984
GBR	2010	1855
GBR	2012	1761
GBR	2014	1895
GBR	2016	1653
GBR	2018	1885
GRC	2009	1651
GRC	2011	1879
HRV	2019	1484
HUN	2009	1020
HUN	2010	1150
HUN	2012	1437
HUN	2015	1203
HUN	2017	1141
HUN	2019	1221

Ccode	Year	n
IRL	2007	1257
IRL	2009	1564
IRL	2011	2063
IRL	2013	2150
IRL	2014	1745
IRL	2017	2084
IRL	2019	1785
ISL	2017	778
ISL	2019	755
ISR	2015	1981
ISR	2016	1961
ITA	2013	697
ITA	2017	1549
ITA	2019	1826
LTU	2011	865
LTU	2013	1250
LTU	2015	1241
LTU	2017	1299
LTU	2019	1053
LVA	2007	1137
LVA	2009	1434
LVA	2019	542
NLD	2008	1608
NLD	2010	1635
NLD	2012	1675
NLD	2014	1682
NLD	2016	1480
NLD	2018	1442
NOR	2008	1480
NOR	2010	1448
NOR	2012	1570
NOR	2014	1364
NOR	2016	1450
NOR	2019	1272
POL	2008	1180
POL	2010	1272
POL	2012	1380
POL	2015	1112
POL	2016	1177
POL	2018	1009

Ccode	Year	n
PRT	2007	1295
PRT	2009	1307
PRT	2011	1259
PRT	2013	1304
PRT	2015	1015
PRT	2017	1082
PRT	2019	845
RUS	2017	1320
SRB	2018	1112
SVK	2008	1321
SVK	2010	1327
SVK	2012	1490
SVK	2019	865
SVN	2008	937
SVN	2010	869
SVN	2012	797
SVN	2014	857
SVN	2016	1012
SVN	2018	1006
SWE	2008	1645
SWE	2010	1348
SWE	2012	1652
SWE	2014	1623
SWE	2016	1384
SWE	2018	1404

total n = 213512

Table 9a:distribution of continuous variables, subsample homophobia

	Mean	SD	Min	Max
economic	5.040	2.438	0.000	10.000
cultural	5.597	2.538	0.000	10.000
place	4.983	2.300	0.000	10.000
Mipex	56.764	14.163	34.000	88.000
dem. satisfaction	5.330	2.491	0.000	10.000
age	48.333	18.082	14.000	95.000
education	12.735	3.963	0.000	30.000
pol. interest	2.519	0.885	1.000	4.000
sub. hhincome	1.982	0.855	1.000	4.000
left-right	5.134	2.232	0.000	10.000
gdp	41792.403	14334.683	11227.966	107634.837
gini index	31.419	4.010	24.400	41.900
effectivity	1.258	0.651	-0.867	2.287
total n = 362656				

Table 9b:distribution of categorical variables, subsample homophobia

		N	%
Homophobia	Non-Homophobic	120054	33.104
	medium	185130	51.048
	Homophobic	57472	15.848
partycloseness	close party	190074	52.412
	no close party	166735	45.976
	nk	5847	1.612
activity	fulltime	136934	37.759
	parttime	18992	5.237
	self employed	25964	7.159
	unemployed	18959	5.228
	other	161807	44.617
gender	female	188099	51.867
	male	174557	48.133
Mig.back	Natives	304906	84.076
	Migrants	57750	15.924
total n = 362656			

Table 9c: Surveys used in subsample homophobia

Ccode	Year	n
ALB	2012	994
AUT	2003	1734
AUT	2005	1763
AUT	2007	1809
AUT	2015	1538
AUT	2016	1747
AUT	2018	2132
BEL	2002	1456
BEL	2004	1548
BEL	2006	1697
BEL	2008	1648
BEL	2011	1588
BEL	2012	1777
BEL	2014	1677
BEL	2016	1678
BEL	2018	1655
BGR	2006	829
BGR	2009	1359
BGR	2011	1544
BGR	2013	1534
BGR	2018	1147
BGR	2021	2256
CHE	2002	1778
CHE	2004	1871
CHE	2006	1647
CHE	2008	1613
CHE	2010	1368
CHE	2012	1370
CHE	2014	1385
CHE	2016	1379
CHE	2018	1343
CHE	2021	1367
CYP	2006	782
CYP	2008	943
CYP	2011	725
CYP	2012	766
CYP	2018	540
CZE	2002	1018
CZE	2004	1919

Ccode	Year	n
CZE	2009	1694
CZE	2011	2007
CZE	2013	1527
CZE	2015	1744
CZE	2016	1931
CZE	2019	2005
CZE	2021	2000
DEU	2003	2625
DEU	2004	2446
DEU	2006	2465
DEU	2008	2461
DEU	2010	2737
DEU	2012	2739
DEU	2014	2861
DEU	2016	2705
DEU	2018	2205
DNK	2002	1342
DNK	2004	1325
DNK	2006	1369
DNK	2008	1505
DNK	2010	1451
DNK	2013	1529
DNK	2014	1407
DNK	2018	1443
ESP	2002	1141
ESP	2004	1307
ESP	2006	1496
ESP	2008	1937
ESP	2011	1616
ESP	2013	1644
ESP	2015	1541
ESP	2017	1600
ESP	2019	1330
EST	2004	1290
EST	2007	975
EST	2009	1173
EST	2010	1403
EST	2012	1798
EST	2014	1602
EST	2016	1691

Ccode	Year	n
EST	2018	1666
EST	2021	1391
FIN	2002	1808
FIN	2004	1854
FIN	2006	1769
FIN	2008	2032
FIN	2010	1727
FIN	2012	2030
FIN	2014	1920
FIN	2016	1821
FIN	2018	1628
FIN	2021	1489
FRA	2006	1824
FRA	2008	1888
FRA	2010	1606
FRA	2013	1810
FRA	2014	1750
FRA	2016	1854
FRA	2018	1743
FRA	2021	1677
GBR	2002	1777
GBR	2004	1634
GBR	2006	1999
GBR	2008	2016
GBR	2010	1903
GBR	2012	1791
GBR	2014	1932
GBR	2016	1683
GBR	2018	1911
GRC	2003	1824
GRC	2005	1837
GRC	2009	1640
GRC	2011	1841
HRV	2009	1002
HRV	2011	1137
HRV	2019	1531
HRV	2021	1299
HUN	2002	1233
HUN	2005	1104
HUN	2006	1064

Ccode	Year	n
HUN	2009	1087
HUN	2010	1220
HUN	2012	1443
HUN	2015	1263
HUN	2017	1183
HUN	2019	1266
HUN	2021	1471
IRL	2003	1509
IRL	2005	1728
IRL	2007	1262
IRL	2009	1573
IRL	2011	2080
IRL	2013	2143
IRL	2014	1790
IRL	2017	2135
IRL	2019	1802
ISL	2005	495
ISL	2012	659
ISL	2017	790
ISL	2019	772
ISL	2021	805
ISR	2002	2072
ISR	2008	1903
ISR	2011	1628
ISR	2012	2005
ISR	2015	2137
ISR	2016	2136
ITA	2003	867
ITA	2006	1203
ITA	2013	712
ITA	2017	1584
ITA	2019	1845
LTU	2011	959
LTU	2013	1304
LTU	2015	1369
LTU	2017	1340
LTU	2019	1153
LTU	2021	1112
LUX	2003	984
LUX	2004	1238

Ccode	Year	n
LVA	2007	1179
LVA	2009	1479
LVA	2019	575
MNE	2019	733
NLD	2002	2185
NLD	2004	1713
NLD	2006	1733
NLD	2008	1651
NLD	2010	1688
NLD	2012	1732
NLD	2014	1742
NLD	2016	1535
NLD	2018	1493
NLD	2021	1367
NOR	2002	1957
NOR	2004	1704
NOR	2006	1679
NOR	2008	1491
NOR	2010	1463
NOR	2012	1588
NOR	2014	1382
NOR	2016	1476
NOR	2019	1305
NOR	2021	1349
POL	2002	1599
POL	2004	1269
POL	2006	1316
POL	2008	1250
POL	2010	1376
POL	2012	1478
POL	2015	1208
POL	2016	1300
POL	2018	1095
PRT	2002	1061
PRT	2005	1247
PRT	2007	1423
PRT	2009	1420
PRT	2011	1296
PRT	2013	1353
PRT	2015	1043

Ccode	Year	n
PRT	2017	1120
PRT	2019	890
PRT	2021	1349
ROU	2006	1355
ROU	2009	1244
RUS	2006	1047
RUS	2008	1249
RUS	2011	1420
RUS	2012	1463
RUS	2017	1347
SRB	2018	1173
SVK	2004	1074
SVK	2006	1397
SVK	2008	1412
SVK	2010	1433
SVK	2012	1555
SVK	2019	884
SVK	2021	1110
SVN	2002	1104
SVN	2004	937
SVN	2006	1001
SVN	2008	961
SVN	2010	878
SVN	2012	808
SVN	2014	878
SVN	2016	1023
SVN	2018	1014
SVN	2020	1030
SWE	2002	1799
SWE	2004	1780
SWE	2006	1739
SWE	2008	1708
SWE	2010	1401
SWE	2012	1708
SWE	2014	1666
SWE	2016	1435
SWE	2018	1440
TUR	2006	1290
TUR	2009	1655
UKR	2005	989

Ccode	Year	n
UKR	2006	955
UKR	2009	760
UKR	2011	797
UKR	2013	982

total n = 362656

Table 10a:distribution of continuous variables, subsample gender

	Mean	SD	Min	Max
Gender equality	0.767	0.101	0.353	0.983
dem. satisfaction	5.310	2.498	0.000	10.000
age	48.549	18.150	14.000	95.000
education	12.679	3.984	0.000	30.000
pol. interest	2.525	0.887	1.000	4.000
sub. hhincome	1.997	0.861	1.000	4.000
left-right	5.138	2.238	0.000	10.000
gdp	41504.915	14420.017	11227.966	107634.837
gini index	31.459	4.041	24.400	41.900
effectivity	1.242	0.660	-0.867	2.287
total n = 372058				

Table 10b:distribution of categorical variables, subsample gender

		N	%
partycloseness	close party	194773	52.350
	no close party	170988	45.957
	nk	6297	1.692
activity	fulltime	139170	37.405
	parttime	19201	5.161
	self employed	26432	7.104
	unemployed	19462	5.231
	other	167793	45.099
gender	female	193174	51.920
	male	178884	48.080
Mig.back	Natives	312851	84.087
	Migrants	59207	15.913
total n = 372058			

Table 10c: Surveys used in subsample gender

Ccode	Year	n
ALB	2012	1030
AUT	2003	1780
AUT	2005	1807
AUT	2007	1848
AUT	2015	1611
AUT	2016	1785
AUT	2018	2184
BEL	2002	1474
BEL	2004	1555
BEL	2006	1699
BEL	2008	1654
BEL	2011	1593
BEL	2012	1784
BEL	2014	1679
BEL	2016	1680
BEL	2018	1661
BGR	2006	913
BGR	2009	1539
BGR	2011	1765
BGR	2013	1714
BGR	2018	1272
BGR	2021	2293
CHE	2002	1816
CHE	2004	1886
CHE	2006	1653
CHE	2008	1621
CHE	2010	1383
CHE	2012	1379
CHE	2014	1394
CHE	2016	1389
CHE	2018	1351
CHE	2021	1380
CYP	2006	814
CYP	2008	981
CYP	2011	757
CYP	2012	791
CYP	2018	551
CZE	2002	1072
CZE	2004	2026

Ccode	Year	n
CZE	2009	1744
CZE	2011	2047
CZE	2013	1599
CZE	2015	1782
CZE	2016	1964
CZE	2019	2051
CZE	2021	2034
DEU	2003	2643
DEU	2004	2458
DEU	2006	2478
DEU	2008	2482
DEU	2010	2759
DEU	2012	2755
DEU	2014	2866
DEU	2016	2711
DEU	2018	2212
DNK	2002	1353
DNK	2004	1333
DNK	2006	1375
DNK	2008	1513
DNK	2010	1455
DNK	2013	1534
DNK	2014	1412
DNK	2018	1447
ESP	2002	1220
ESP	2004	1335
ESP	2006	1506
ESP	2008	1968
ESP	2011	1633
ESP	2013	1656
ESP	2015	1575
ESP	2017	1620
ESP	2019	1349
EST	2004	1382
EST	2007	1034
EST	2009	1222
EST	2010	1448
EST	2012	1858
EST	2014	1636
EST	2016	1692

Ccode	Year	n
EST	2018	1670
EST	2021	1393
FIN	2002	1824
FIN	2004	1861
FIN	2006	1773
FIN	2008	2033
FIN	2010	1734
FIN	2012	2039
FIN	2014	1932
FIN	2016	1828
FIN	2018	1634
FIN	2021	1496
FRA	2006	1829
FRA	2008	1896
FRA	2010	1613
FRA	2013	1815
FRA	2014	1754
FRA	2016	1865
FRA	2018	1750
FRA	2021	1686
GBR	2002	1782
GBR	2004	1642
GBR	2006	2009
GBR	2008	2027
GBR	2010	1915
GBR	2012	1809
GBR	2014	1940
GBR	2016	1687
GBR	2018	1916
GRC	2003	1932
GRC	2005	1916
GRC	2009	1677
GRC	2011	1923
HRV	2009	1046
HRV	2011	1191
HRV	2019	1550
HRV	2021	1325
HUN	2002	1327
HUN	2005	1175
HUN	2006	1179

Ccode	Year	n
HUN	2009	1170
HUN	2010	1294
HUN	2012	1599
HUN	2015	1342
HUN	2017	1252
HUN	2019	1312
HUN	2021	1524
IRL	2003	1547
IRL	2005	1769
IRL	2007	1297
IRL	2009	1583
IRL	2011	2128
IRL	2013	2190
IRL	2014	1821
IRL	2017	2148
IRL	2019	1820
ISL	2005	499
ISL	2012	662
ISL	2017	793
ISL	2019	775
ISL	2021	810
ISR	2002	2082
ISR	2008	2061
ISR	2011	1726
ISR	2012	2119
ISR	2015	2249
ISR	2016	2221
ITA	2003	878
ITA	2006	1213
ITA	2013	722
ITA	2017	1615
ITA	2019	1882
LTU	2011	996
LTU	2013	1411
LTU	2015	1452
LTU	2017	1433
LTU	2019	1185
LTU	2021	1123
LUX	2003	1021
LUX	2004	1255

Ccode	Year	n
LVA	2007	1236
LVA	2009	1553
LVA	2019	597
MNE	2019	738
NLD	2002	2191
NLD	2004	1718
NLD	2006	1739
NLD	2008	1653
NLD	2010	1692
NLD	2012	1736
NLD	2014	1745
NLD	2016	1540
NLD	2018	1499
NLD	2021	1369
NOR	2002	1961
NOR	2004	1709
NOR	2006	1680
NOR	2008	1492
NOR	2010	1469
NOR	2012	1591
NOR	2014	1385
NOR	2016	1480
NOR	2019	1311
NOR	2021	1352
POL	2002	1680
POL	2004	1326
POL	2006	1361
POL	2008	1295
POL	2010	1439
POL	2012	1545
POL	2015	1241
POL	2016	1349
POL	2018	1134
PRT	2002	1122
PRT	2005	1301
PRT	2007	1489
PRT	2009	1501
PRT	2011	1376
PRT	2013	1436
PRT	2015	1055

Ccode	Year	n
PRT	2017	1125
PRT	2019	902
PRT	2021	1389
ROU	2006	1415
ROU	2009	1340
RUS	2006	1184
RUS	2008	1422
RUS	2011	1536
RUS	2012	1652
RUS	2017	1427
SRB	2018	1210
SVK	2004	1121
SVK	2006	1435
SVK	2008	1486
SVK	2010	1501
SVK	2012	1590
SVK	2019	925
SVK	2021	1157
SVN	2002	1126
SVN	2004	962
SVN	2006	1034
SVN	2008	989
SVN	2010	910
SVN	2012	851
SVN	2014	907
SVN	2016	1039
SVN	2018	1030
SVN	2020	1043
SWE	2002	1820
SWE	2004	1790
SWE	2006	1757
SWE	2008	1719
SWE	2010	1409
SWE	2012	1712
SWE	2014	1668
SWE	2016	1439
SWE	2018	1445
TUR	2006	1423
TUR	2009	1809
UKR	2005	1150

Ccode	Year	n
UKR	2006	1070
UKR	2009	930
UKR	2011	914
UKR	2013	1132

total n = 372058
